# PARP3 supervises G9a-mediated repression of adhesion and hypoxia-responsive genes in glioblastoma cells

**DOI:** 10.1038/s41598-022-19525-6

**Published:** 2022-09-15

**Authors:** Leonel Nguekeu-Zebaze, Najat Hanini, Aurélia Noll, Nadège Wadier, Jean-Christophe Amé, Lisa Roegel, Françoise Dantzer

**Affiliations:** Poly(ADP-Ribosyl)Ation and Genome Integrity, Strasbourg Drug Discovery and Development Institute (IMS), UMR7242, Centre Nationale de la Recherche Scientifique/Université de Strasbourg, Institut de Recherche de l’Ecole de Biotechnologie de Strasbourg, 300 Bld. S. Brant, CS10413, 67412 Illkirch, France

**Keywords:** Cancer, Cell biology, Diseases, Oncology

## Abstract

In breast cancer, Poly(ADP-ribose) polymerase 3 (PARP3) has been identified as a key driver of tumor aggressiveness exemplifying its selective inhibition as a promising surrogate for clinical activity onto difficult-to-treat cancers. Here we explored the role of PARP3 in the oncogenicity of glioblastoma, the most aggressive type of brain cancer. The absence of PARP3 did not alter cell proliferation nor the in vivo tumorigenic potential of glioblastoma cells. We identified a physical and functional interaction of PARP3 with the histone H3 lysine 9 methyltransferase G9a. We show that PARP3 helps to adjust G9a-dependent repression of the adhesion genes *Nfasc* and *Parvb* and the hypoxia-responsive genes *Hif-2α*, *Runx3*, *Mlh1*, *Ndrg1, Ndrg2* and *Ndrg4*. Specifically for *Nfasc*, *Parvb* and *Ndrg4*, PARP3/G9a cooperate for an adjusted establishment of the repressive mark H3K9me2. While examining the functional consequence in cell response to hypoxia, we discovered that PARP3 acts to maintain the cytoskeletal microtubule stability. As a result, the absence of PARP3 markedly increases the sensitivity of glioblastoma cells to microtubule-destabilizing agents providing a new therapeutic avenue for PARP3 inhibition in brain cancer therapy.

## Introduction

Poly(ADP-ribose) polymerase 3 (PARP3) is a member of the PARP family that catalyzes mono-ADP-ribosylation (or MARylation), the transfer of a single ADP-ribose molecule onto itself, a number of other protein substrates or on terminal phosphate residues at double- and single-strand break termini of DNA molecules^1–5^. PARP3 was originally identified as a key regulator of the classical non-homologous end-joining pathway (C-NHEJ) for the repair of double-strand breaks, and as an important protein securing telomeric segregation during mitosis^3–5^. Subsequently, PARP3 has been proposed to mediate the ADP-ribosylation of chromatin at sites of single strand breaks, to promote chromosomal rearrangements and limit G4 DNA and to stimulate breast tumor aggressiveness exemplifying its selective inhibition as a promising therapeutic strategy to treat highly aggressive cancers^6–9^. Accumulating studies also suggest emerging roles of PARP3 in the regulation of gene expression mediated by its interaction with chromatin regulators. In humans, PARP3 was reported to associate with several Polycomb group proteins (PcG) of the PRC2 complex namely EZH2, SUZ12, RbAp46/48, YY1 and HDAC1/2, key transcriptional regulators of embryogenesis and development^10^. In line with this, PARP3 was described to have pivotal functions in ectodermal specification and neural crest development in the Zebrafish by regulating the transcriptional expression of key transcription factors^11^. In glioblastoma cells, PARP3 was proposed to enhance the transcriptional activity of FOXM1 to confer glioblastoma cell radioresistance^12^.

G9a (also known as EHMT2) and the closely related GLP1 (EHMT1) are SET-domain containing lysine methyltransferases that mono- (for GLP1) and di-methylate (for G9a) respectively histone 3 lysine 9 in euchromatin (H3K9me1/me2) to exert transcriptional silencing. They have also been shown to methylate histones H1^13^, H3K27^14,15^ and H3K56^16^ as well as a number of non histone proteins. Similarly to PARP3, although with distinct functions, G9a in complex or not with GLP1, has been shown to mediate the repair of double-strand breaks, to play critical roles in development and to promote cancer progression. Often G9a exerts these biological activities through its association with key partners. To support gene repression, the G9a/GLP1 heterodimer interacts with a third complex member, the Widely Interspaced Zinc Finger (WIZ). WIZ regulates global H3K9me2 levels by promoting G9a and GLP1 protein stability^17^, and by facilitating the retention of G9a at chromatin and specific loci in cooperation with the zinc finger protein ZNF644^18,19^. The G9a/GLP1 complex also interacts and cooperates with a subset of other SET-domain containing proteins including SETDB1 and Suv39h1 to establish and maintain the silencing of major satellite repeats and few G9a-targets^20^ and with PRC2 to mediate its genomic recruitment to specific common target genes encoding developmental and neuronal regulators^21,22^. Following double-strand breaks, G9a is recruited to chromatin and damaged DNA as a result of ATM and/or casein kinase 2-mediated phosphorylation^23,24^. While G9a activity contributes to the early recruitment of the repair proteins BRCA1 and 53BP1 to DNA breaks^23^, its interaction with RPA permits efficient homologous recombination-driven repair^24^. Similarly to PARP3, G9a has been associated with tumor aggressiveness. G9a is frequently overexpressed in several tumor types and its absence has been shown to reduce tumor growth and metastasis^25,26^. In the context of hypoxia, a key factor of tumor strength, G9a has been identified as a mediator of hypoxia-dependent gene repression either by catalyzing H3K9me2 or by modulating the expression or the activity of the master transcriptional regulator hypoxia-inducible factors (HIF)^27–30^.

Here we aimed to decipher the functional role of PARP3 in the oncogenicity of glioblastoma, the most aggressive primary brain cancer. We identified a physical and functional interaction of PARP3 with G9a. PARP3 ADP-ribosylates G9a in vitro. We show that in the model of glioblastoma, G9a and PARP3 functionally cooperate to regulate the expression of the adhesion genes *Nfasc* and *Parvb* and the hypoxia-responsive genes *Mlh1*, *Hif-2α*, *Runx3*, *Ndrg1, Ndrg2* and *Ndrg4*. While exploring the outcome in the hypoxic response, we uncovered a contribution of PARP3 in the cytoskeleton microtubules stability. In consequence, the absence of PARP3 sensitizes glioblastoma cells to microtubule destabilizing anti-cancer agents.

## Results

### PARP3 deficiency has no impact on cell proliferation nor tumor growth of glioblastoma cell lines

**Figure 1:**
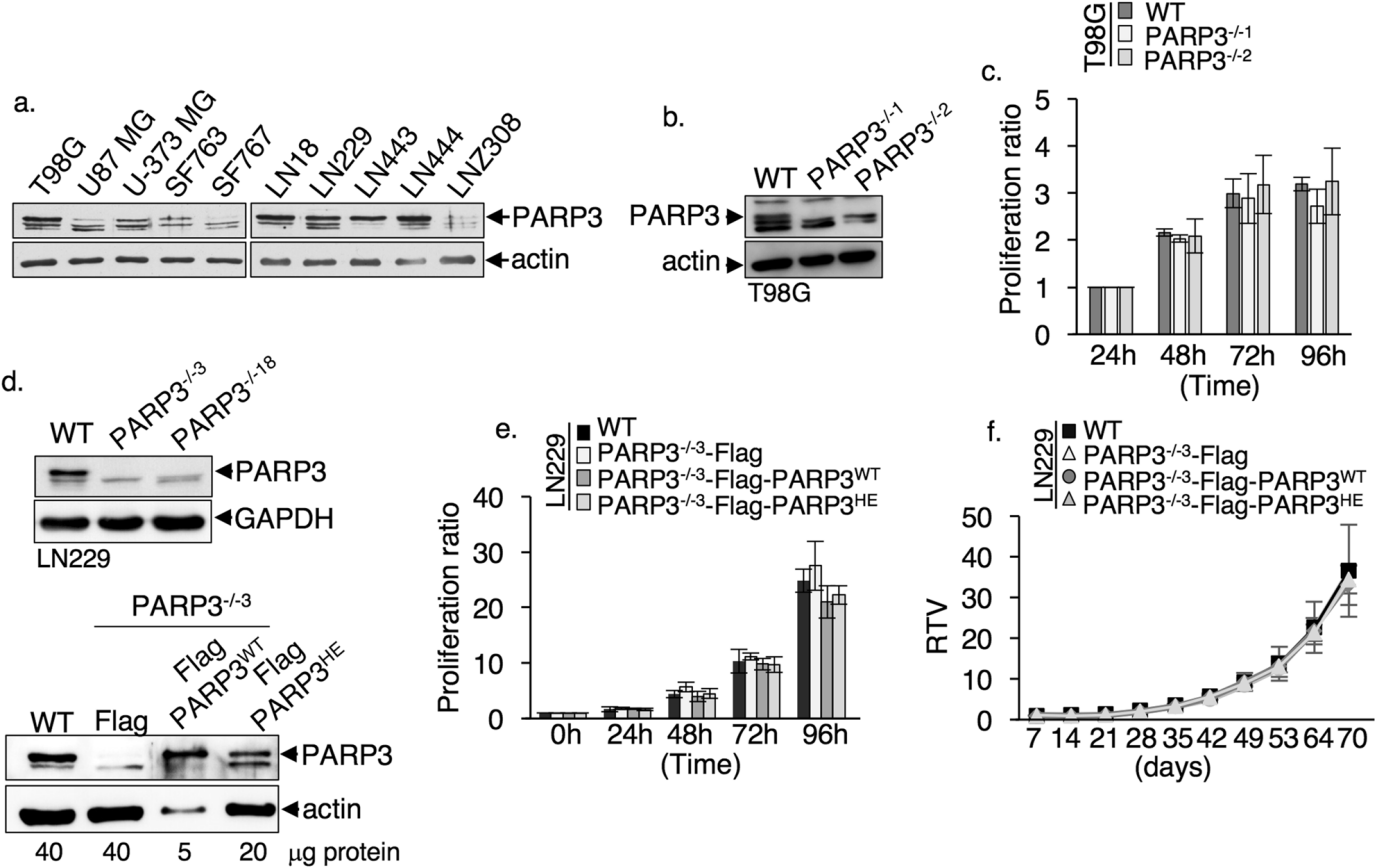
The absence of PARP3 has no impact on cell proliferation and tumor growth in LN229 and T98G cells. (**a**) PARP3 is highly expressed in glioblastoma cell lines. The protein expression levels of PARP3 and actin were examined on nuclear protein extracts and by western blotting using the appropriate antibodies. Original blots are shown in the supplementary information file. (**b**) Western blot analysis of PARP3 and actin expression in the wild-type (WT) and two PARP3^−/−1^, PARP3^−/−2^ T98G clones selected upon screening and sequence analysis. Uncropped Western-blots are shown in the supplementary information file. (**c**) Graphs compare proliferation rates between the parental T98G (WT) and the PARP3^−/−1^ and PARP3^−/−2^ T98G cells. Experiments were performed three times. Mean values of triplicates ± s.d are indicated. (**d**) *Upper panel*, Western blot analysis of PARP3 and GAPDH expression in the wild-type (WT) and two PARP3^−/−3^, PARP3^−/−18^ LN229 clones selected upon screening and sequence analysis. *Lower panel,* Western blot analysis of PARP3 and actin expression in the wild-type (WT) and the PARP3^−/−3^ LN229 cells with a stable expression of either the Flag control (Flag), Flag-PARP3^WT^ or Flag-PARP3^HE^. Loading amounts that have been optimized for the figure are indicated. Uncropped Western-blots are shown in the supplementary information file. (**e**) Graphs compare proliferation rates between the parental LN229 (WT) and the PARP3^−/−3^ LN229 cell lines expressing either the Flag control (Flag), Flag-PARP3^WT^ or Flag-PARP3^HE^ fusion proteins. Experiments were performed three times giving similar results. Mean values of triplicates ± s.d. of a representative experiment are indicated. (**f**) Relative tumor growth curves of xenografts derived from the wild type (WT) and the PARP3^−/−3^ LN229 cell lines expressing either the Flag control (Flag), Flag-PARP3^WT^ or Flag-PARP3^HE^ fusion proteins. Mean RTV ± s.d. (n = 6 individual mice) are expressed compared to tumor volumes on day 7 for all cell lines.

Given the increasing appreciation for the role of PARP3 in tumor aggressiveness^6,9,31^, we aimed to investigate whether PARP3 might be involved in the pathogenesis of glioblastoma, one of the most malignant and aggressive brain cancer in adults. We first compared the expression of PARP3 in a series of human brain cancer cell lines. We found that PARP3 expression is predominant in all but not in two (U87MG, SF767) glioblastoma cell lines and poorly detectable in the astrocytoma cell line LNZ308 (Fig. 1a). Next, we inactivated PARP3 in LN229 and T98G cells using the double nCas9(D10A) strategy^6^. For each cell line two independent PARP3^−/−^ clones were isolated (Fig. 1b,d and Sup. Fig. 1). While the absence of PARP3 or its inactivation alters the tumorigenicity of the highly aggressive BRCA1-deficient TNBC cells^6^, its disruption had no impact on the proliferation rate of LN229 nor T98G cells (Fig. 1c,e). To consider the functional importance of PARP3’s catalytic activity, we also re-expressed Flag-PARP3 (Flag-PARP3^WT^), a catalytically inactive mutant (Flag-PARP3^HE^), or a Flag peptide alone as control in the PARP3^−/−3^ LN229 cells (Fig. 1d). The expression of the catalytically inactive mutant did not more compromise the proliferation rate of the LN229 cells (Fig. 1e) nor their tumorigenic potential when subcutaneously xenografted into nude mice (Fig. 1f).

### PARP3 interacts with the chromatin repressive complex G9a/GLP1/Wiz and ADP-ribosylates G9a

**Figure 2:**
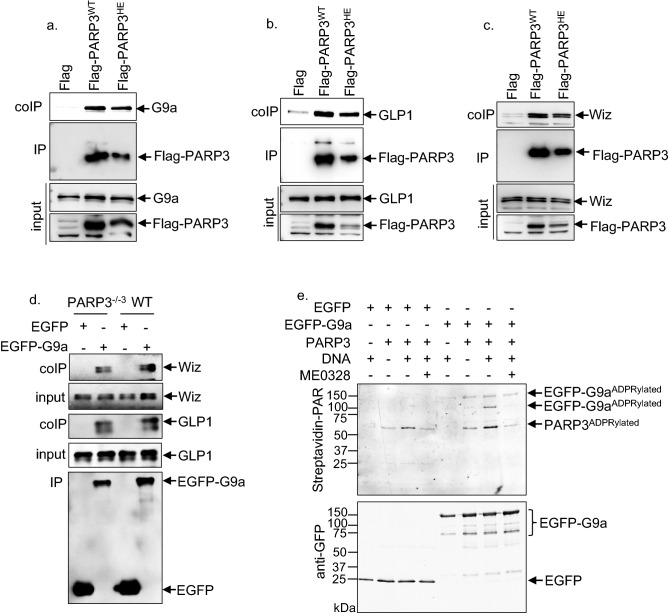
PARP3 interacts with and ADP ribosylates G9. (**a–c**) G9a, GLP1 and Wiz co-immunoprecipitate with Flag-PARP3^WT^ and Flag-PARP3^HE^ but not with Flag. The PARP3^−/−3^-Flag, PARP3^−/−3^-Flag-PARP3^WT^ and PARP3^−/−3^-Flag-PARP3^HE^ LN229 cell extracts were immunoprecipitated with a Flag antibody and analysed by western blotting using anti-G9a (**a**), anti-GLP1 (**b**), anti-Wiz (**c**) and anti-Flag antibodies. Input corresponds to 1/40 of the total amount of cell extracts used for immunoprecipitation. (**d**) The absence of PARP3 does not alter the stability of the G9a/GLP1/Wiz complex. The parental (WT) and the PARP3^−/−3^ LN229 cells were transfected with either EGFP (control) or EGFP-G9a. EGFP and EGFP-G9a immunoprecipitates were analysed by western blotting using anti-Wiz, anti-GLP1 and anti-EGFP antibodies. Input corresponds to 1/30 of the total amount of cell extracts used for immunoprecipitation. (**e**) PARP3 ADP-ribosylates G9a in vitro. Immunopurified EGFP (control) or EGFP-G9a were incubated with purified PARP3 in the absence or in the presence of DNase-activated DNA and biotinylated NAD^+^. When indicated ME0328 (20 µM) was added in the reaction buffer to inhibit PARP3 catalytic activity. ADP-ribosylated G9a and PARP3 (auto-ADP-ribosylated) are detected using the streptavidin Alexa system. EGFP and EGFP-G9a are detected by western blotting using an anti-GFP antibody. Uncropped Western-blots are shown in the supplementary information file.

Next, we decided to search for PARP3-specific protein partners. In an initial mass spectrometry analysis of the proteins interacting with Flag-PARP3 immunoprecipitates performed in the PARP3^−/−3^-Flag-PARP3^WT^ LN229 whole cell extracts, we captured the chromatin repressive complex G9a/GLP1/Wiz (data not shown)*.* Given the functional cooperation of G9a/GLP1 with PRC2^21^, a binding partner of PARP3^10^, we decided to validate this association further and question on the importance of PARP3’s catalytic activity. To this end, we performed independent immunoprecipitations of Flag-PARP3^WT^, Flag-PARP3^HE^ and Flag as a control using an anti-Flag antibody and tested for the presence of G9a, GLP1 and Wiz using the appropriate antibodies (Fig. 2a–c). To prevent from unspecific association mediated by DNA, cell lysates were pretreated with DNase I before immunoprecipitation. We confirmed efficient co-precipitation of each G9a, GLP1 and Wiz with both Flag-PARP3^WT^ and Flag-PARP3^HE^ while only a minor to no association with Flag alone was detected. These results suggested that the association of PARP3 with the G9a/GLP1/Wiz complex does not involve its catalytic activity nor its binding to DNA. We further validated the co-precipitation of G9a and Wiz with EGFP-PARP3 immunoprecipitates in HEK cells while no co-precipitation was detected with EGFP (Sup. Fig. 2a). We also performed reciprocal immunoprecipitations in the PARP3^−/−3^-Flag-PARP3^WT^ LN229 cells using EGFP-G9a as a bait. We confirmed Flag-PARP3^WT^ as a binding partner of EGFP-G9a while no interaction was detected in the EGFP immunoprecipitates (Sup. Fig. 2b). To verify the role of PARP3 in the stability of the complex, we then studied the co-precipitation of Wiz and GLP1 with EGFP-G9a or EGFP used as control expressed in WT LN229 versus PARP3^−/−3^ LN229 cells (Fig. 2d). Relative to the levels of EGFP-G9a immunoprecipitates, we detected a similar co-purification of Wiz and GLP1 in either WT or PARP3-deficient cells indicating that PARP3 does not regulate the strength of the complex. No association with EGFP alone was revealed. To substantiate on this association, we next evaluated the ability of PARP3 to ADP-ribosylate G9a (Fig. 2e). Immunopurified EGFP-G9a or EGFP alone as a control was incubated together with purified PARP3 in the presence of biotinylated NAD^+^ and in the absence or the presence of DNase-I-treated calf thymus DNA to enhance PARP3 activity. As an additional control, we used the PARP3 inhibitor ME0328. The expression of EGFP-G9a produces different migrating bands interpreted as the full-length protein and truncated variants. Considering that G9a binds DNA with high affinity, the possible co-purification of a weak amount of DNA in the EGFP-G9a immunoprecipitates is possible and explains the automodification of PARP3 and the ADP-ribosylation of the upper band of EGFP-G9a. The addition of fragmented DNA in the reaction buffer stimulates the automodification of PARP3 and reveals the ADP-ribosylation of other accessible residues in the truncated EGFP-G9a that can be hidden in the full length protein because of a different structural folding. No ADP-ribosylation of EGFP alone was detected. With a different degree, the modification of both variants was reduced in the presence of ME0328 confirming the PARP3-catalysed modification. Together, these results reveal that PARP3 is able to directly ADP-ribosylate G9a.

### PARP3 deficiency alters the expression of clusters of genes involved in cell adhesion, cell migration and cell communication involving the ECM

**Figure 3:**
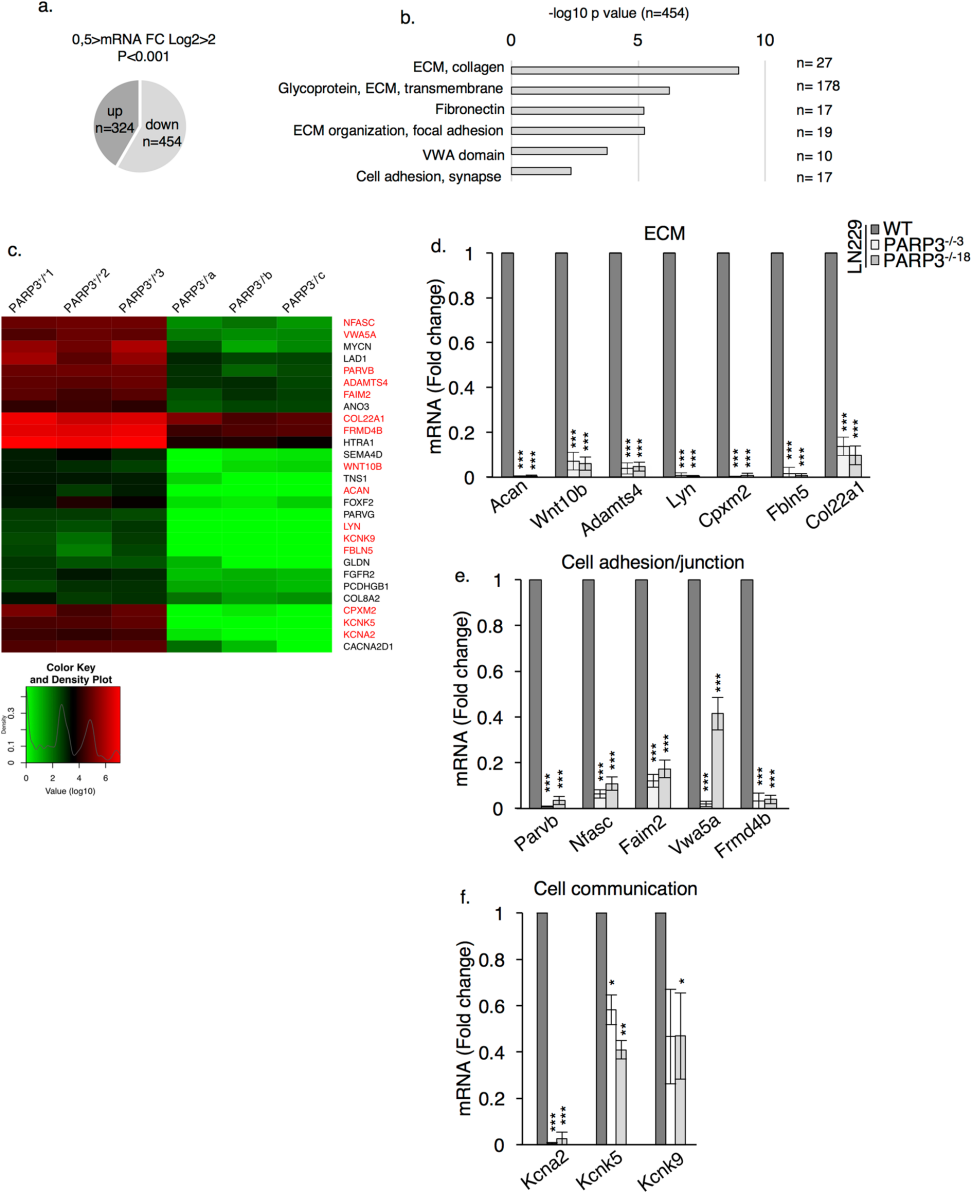
RNA seq reveals downregulation of transcripts coding for proteins involved in the organization of the ECM, in cell adhesion/junction, cell migration and communication. (**a**) Venn diagram illustrating the number of deregulated transcripts in PARP3^−/−^ LN229 cells (454 downregulated genes and 324 upregulated genes). (**b**) Pathway overrepresentation analysis of the 454 downregulated transcripts deduced with the DAVID bioinformatics resources 6.8. Number in brackets indicate the number of genes enriched, clustering to the corresponding pathway. (**c**) Clustered heatmap of downregulated transcripts in the ECM, cell adhesion and communication pathways. PARP3^+/+1^, PARP3^+/+2^, PARP3^+/+3^ represent three biological replicates of WT RNA extracts while PARP3^−/−a^, PARP3^−/−b^, PARP3^−/−c^ represent three biological replicates of PARP3-deficient RNA extracts. Transcripts highlighted in red were used for subsequent validation experiments. (**d–f**) qPCR validation of the selected downregulated transcripts from the ECM (**d**), cell adhesion/junction (**e**) and cell communication (**f**) pathways in WT and PARP3^−/−3^ and PARP3^−/−18^ LN229 cell lines. Data are expressed relative to *Gapdh*. Values represent means ± s.e.m. of three independent experiments and three technical replicates. *p < 0.05, **p < 0.01,***p < 0.001.

To further decrypt the functions of PARP3 in glioblastoma and identify gene regulatory pathways controlled by PARP3, we performed a comparative RNAseq of the WT-LN229 and PARP3^−/−^ LN229 cells. This approach identified 454 downregulated genes and 324 upregulated genes (Fig. 3a) in the PARP3^−/−^ LN229 cells versus the WT LN229 cells. A pathway overrepresentation analysis of the downregulated transcripts using the DAVID interface revealed substantial changes in pathways and molecules associated with the ECM composition and function including collagen and glycoproteins and with cell adhesion and junctions and cell communication including channel activity (Fig. 3b). A clustered heatmap of a panel of selected transcripts from these groups and the validation of several of these genes by qPCR analysis confirmed that the absence of PARP3 significantly downregulated the selected transcripts consistently in the three independent experiments (Fig. 3c–f). Among those, 2 genes belong to the top 10/454 most significantly downregulated genes (*Nfasc*, *Frmd4b*) and 2 others were classified in the top 20/454 most significantly downregulated genes (*Adamts4*, *Vwa5a*).

### PARP3 modulates G9a-mediated repression of the adhesion genes *Nfasc* and *Parvb*

**Figure 4:**
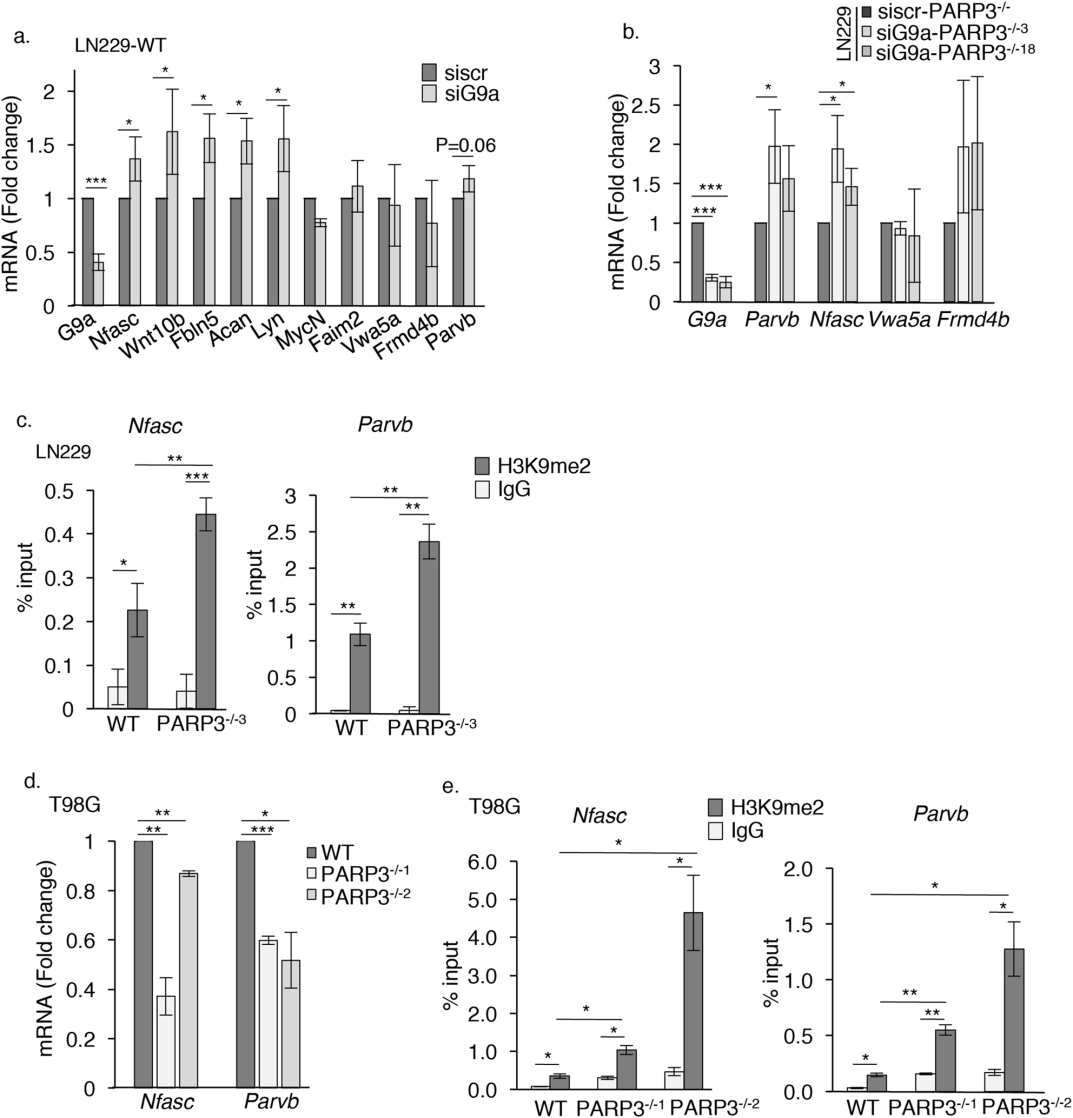
PARP3 moderates the G9a-mediated repression of the adhesion genes *Nfasc* and *Parvb.* (**a**) The silencing of G9a increases the expression of *Nfasc*, *Wnt10b*, *Fbln5*, *Acan*, *Lyn*. The parental LN229 cells were transfected with either sicontrol or siG9a for 72 h. The ECM (*Wnt10b*, *Fbln5*, *Acan*, *Lyn*), adhesion/junction (*Nfasc*, *Faim2*, *Vwa5a*, *Frmd4b*, *Parvb*) and *G9a* mRNA levels were analysed by RT-qPCR. Data are expressed relative to *Gapdh*. Values represent means ± s.e.m. of 3 independent experiments and 3 technical replicates. *p < 0.05, ***p < 0.001. (**b**) Recovery of expression of *Parvb* and *Nfasc* in the PARP3-deficient cells upon G9a silencing. The PARP3^−/−3^ and PARP3^−/−18^ LN229 cells were transfected with either sicontrol or siG9a for 72 h. The transcript levels of *G9a*, *Parvb*, *Nfasc*, *Frmd4b* and *Vwa5a* were analysed by RT-qPCR. Data are expressed relative to *Gapdh*. Values represent means ± s.e.m of 3 independent experiments and 3 technical replicates. *p < 0.05, ***p < 0.001 (**c**) Increased enrichment of H3K9me2 on the promoters of *Nfasc* and *Parvb* genes in PARP3-deficient LN229 cells. H3K9me2 binding was assessed on the promoter regions of *Nfasc*, *Parvb* by CHIP-qPCR in the parental WT versus the PARP3^−/−3^ LN229 cells. IgG were used as ChIP negative controls. Data are represented as percent of input and are means (± s.e.m) of three biological replicates. *p < 0.05, **p < 0.01, ***p < 0.001. (**d**) Repression of *Nfasc* and *Parvb* in PARP3-deficient T98G cells. The transcript levels of *Parvb* and *Nfasc* were analysed by RT-qPCR in WT, and PARP3^−/−1^ and PARP3^−/−2^ T98G cell lines. Data are expressed relative to *Actin*. Values represent means ± s.e.m of 3 independent experiments and 3 technical replicates. *p < 0.05, **p < 0.01, ***p < 0.001. (**e**) Increased enrichment of H3K9me2 on the promoters of *Nfasc* and *Parvb* genes in PARP3-deficient T98G cells. H3K9me2 binding was assessed on the promoter regions of *Nfasc*, *Parvb* by ChIP-qPCR in the parental WT versus the two PARP3^−/−1^ and PARP3^−/−2^ T98G cells. IgG were used as ChIP negative controls. Data are represented as percent of input and are means (± s.e.m) of three biological replicates. *p < 0.05, **p < 0.01.

Since G9a has previously been reported to mediate transcriptional repression of ECM and adhesion genes, we searched for a possible functional interplay between PARP3 and G9a in the regulation of candidate genes from these groups. To identify commonly regulated genes, we first analysed the impact of G9a silencing in WT LN229 cells, on the expression of 10 selected genes that we found repressed in the PARP3 knockout LN229 cells (Fig. 4a). We detected a significant upregulation in the expression of the ECM-related genes *Wnt10b*, *Fbln5*, *Acan* and *Lyn* and the adhesion-related genes *Nfasc* and a mild upregulation of the adhesion-related gene *Parvb* following the depletion of G9a in the WT LN229 cells. These results indicated that the expression of those genes are inversely regulated by PARP3 and G9a. The expression of some of these genes were then analysed in the PARP3-deficient cells upon G9a silencing (Fig. 4b). Among the 4 genes down-regulated in the absence of PARP3, the two adhesion genes *Nfasc* and *Parvb* appeared no longer repressed upon G9a knockdown. The expression of *Nfasc* significantly increased in the siG9a versus the sicontrol in both PARP3^−/−3^ and PARP3^−/−18^ LN229 cell lines, while a significant upregulation of *Parvb* was predominantly detected in the PARP3^−/−3^ clone. In contrast, *Vwa5a* remained down-regulated following G9a silencing and the upregulation of *Frmd4b* was not significant, suggesting that their repression in the PARP3-deficient cells occur in a G9a-independent manner. These results suggested that PARP3 and G9a conversely co-regulate the expression of *Nfasc* and *Parvb* genes. The major function of the histone methyltransferases G9a is to catalyse H3K9me2 for transcriptional repression^32,33^. To analyse the impact of PARP3 on G9a activity, we then compared the enrichment of H3K9me2 on the promoters of *Nfasc* and *Parvb* genes in the WT versus the PARP3^−/−3^ LN229 cells by ChIP-qPCR (Fig. 4c). We detected a significant increase of H3K9 dimethylation on the promoter of *Nfasc* and *Parvb* genes in the absence of PARP3. To strenghten these results, we verified the repression of *Nfasc* and *Parvb* and the increased enrichment of H3K9me2 on their promoters in the two PARP3-deficient T98G cell lines compared to the WT T98G counterpart (Fig. 4d,e). Notably, the enrichment of H3K9me2 on both genes was reduced in the PARP3^−/−3^ LN229 cells treated with the selective G9a inhibitor UNC0638 and in the PARP3^−/−2^ T98G cells upon G9a silencing (Sup. Fig. 3). Moreover, we detected a notable enrichment of G9a on both *Nfasc* and *Parvb* in the PARP3^−/−3^ LN229 cells (Sup. Fig. 7).

Collectively, the results collected so far indicate that PARP3 interacts with and moderates G9a catalyzed H3K9me2 at the gene specific level onto *Nfasc* and *Parv*b to promote their expression.

### PARP3 modulates G9a-mediated repression of hypoxia-responsive genes

**Figure 5:**
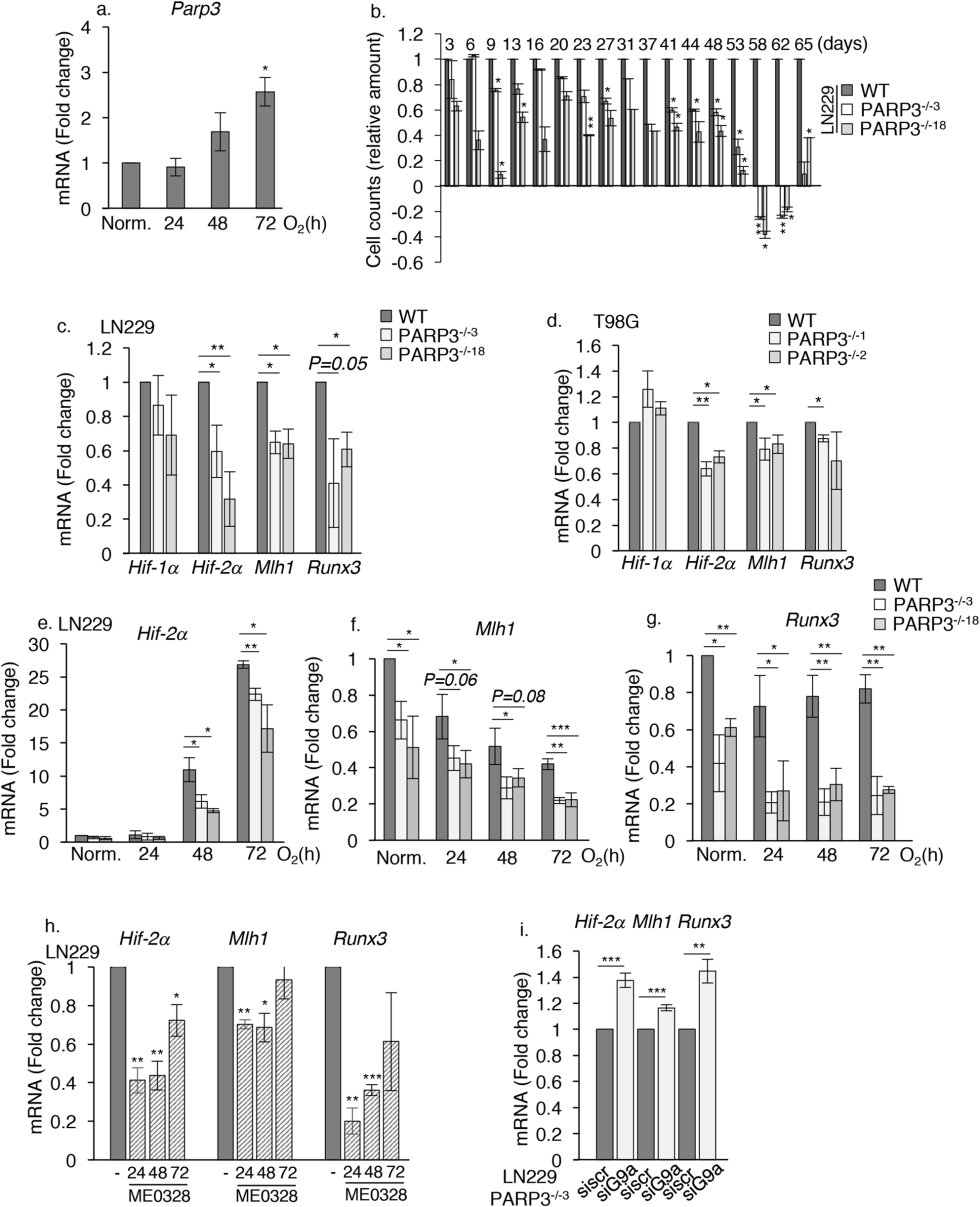
PARP3 mediates cell response to hypoxia in LN229 and counteracts the expression of the G9a-controlled hypoxia-responsive genes *Hif-2α*, *Mlh1* and *Runx3*. (**a**) *Parp3* expression increases during 1% O_2_-induced hypoxia. RT-qPCR analysis of *Parp3* cultured in normoxia (Norm) and at different times upon 1% O_2_. Data are expressed relative to *Actin*. Values represent means ± s.e.m. of 3 independent experiments and 3 technical replicates. *p < 0.05. (**b**) The absence of PARP3 causes susceptibility to chronic chemical-induced hypoxia. The parental WT, PARP3^−/−3^ and PARP3^−/−18^ LN229 cells were maintained under CoCl_2_-induced hypoxia during 65 days. The remaining cells were counted at the indicated time points. Values represent means (± s.e.m) of technical replicates. *p < 0.05, **p < 0.01. (**c-g**) PARP3 deficiency induce the selective downregulation of *Hif-2α*, *Mlh1* and *Runx3* in normoxia and during hypoxia*.* (**c**,**d**) RT-qPCR analysis of *Hif-1α*, *Hif-2α*, *Mlh1* and *Runx3* in the parental WT, and two PARP3-deficient clones of LN229 and T98G cells grown under normal conditions. Data are expressed relative to *Actin*. Values represent means ± s.e.m. of 3 independent experiments and 3 technical replicates. *p < 0.05, **p < 0.01. (**e–g**) RT-qPCR analysis of *Hif-2α*, *Mlh1* and *Runx3* in the parental WT, PARP3^−/−3^ and PARP3^−/−18^ LN229 cells grown under 1% O_2_ for the indicated time points. Data are expressed relative to *Actin*. Values represent means ± s.e.m. of 3 independent experiments and 3 technical replicates. *p < 0,05, **p < 0,01, ***p < 0,001. (**h**) ME0328-mediated chemical inhibition of PARP3 reproduces the selective downregulation of *Hif-2α, Mlh1* and *Runx3* in normoxia. RT-qPCR analysis of *Hif-2α*, *Mlh1* and *Runx3* in the parental WT LN229 cells grown in normoxia in the absence or in the presence of ME0328 for the indicated time points. Data are expressed relative to *Actin*. Values represent means ± s.e.m. of 3 independent experiments and 3 technical replicates. *p < 0.05, **p < 0.01,***p < 0.001. (**i**) Recovery of expression of *Hif-2α*, *Mlh1* and *Runx3* in the PARP3-deficient cells upon G9a silencing. The PARP3^−/−3^ LN229 cells were transfected with either sicontrol or siG9a for 72 h. The transcript levels of *Hif-2α*, *Mlh1* and *Runx3* were analysed by RT-qPCR. Data are expressed relative to *Actin*. Values represent means ± s.e.m. of 3 independent experiments and 3 technical replicates. **p < 0.01; ***p < 0.001.

Several studies have linked G9a to hypoxic regulation^27–30,34^. Earlier, we uncovered an important role of PARP3 in cell response to hypoxia in neural stem progenitor cells^35^. Thus, we aimed to investigate a possible functional cooperation between both partners in this context. First, to ascertain the importance of PARP3 in the hypoxic response, we analysed the effect of 1% O_2_-induced hypoxia on the expression of *Parp3*. We detected a gradual upregulation of *Parp3* from 48 h upon hypoxia (Fig. 5a). We then examined the sensitivity of the PARP3^−/−^ LN229 cells to chronic hypoxia compared to the parental WT cells (Fig. 5b). We submitted our cell models to a 2 months-long exposure to the chemical inducer of hypoxia CoCl_2_, mimicking permanent hypoxia from tumors, and we measured sensitivity throughout time by counting cells at each trypsinization. The absence of PARP3 clearly increased the susceptibility of LN229 cells to a long-term exposure to CoCl_2_. Together, these results suggested that PARP3 mediates long-term response to hypoxia in LN229.

Next, to identify common regulated genes, we analysed the expression of *Hif-1α, Hif-2α, Mlh1* and *Runx3* in our PARP3-deficient cell models and upon PARP3 inhibition because these genes have previously been shown to be directly regulated by G9a under hypoxia^29,30,34^ (Fig. 5c–h). We validated the role of G9a in the downregulation of *Hif-2α* and *Runx3* in the LN229 cells as shown by their upregulation when cells are treated with the G9a catalytic inhibitor UNC0638 (Sup. Fig. 4a). In both LN229 and T98G cells, the expression level of *Hif-1α *was not significantly affected by the absence of PARP3, but we found a selective down-regulation of *Hif-2α*, *Mlh1* and *Runx3* in normoxia (Fig. 5c,d). These repression were maintained in the PARP3-deficient LN229 cells maintained under 1% O_2_ and in the parental WT LN229 cells treated with the PARP3 inhibitor ME0328 when compared to the mock-treated WT cells (Fig. 5e–h) though not in the T98G models exposed to 1%O_2_ for the same duration (Sup. Fig. 4b–d).

To verify whether the repression of these genes in normoxia were mediated by G9a, we measured their expression in the PARP3-deficient LN229 cells upon G9a silencing. The three genes were no more downregulated but upregulated in the PARP3-deficient cells upon G9a silencing (Fig. 5i). *Hif-2α* was also upregulated in the G9a-depleted PARP3^−/−18^ cells (Sup. Fig. 4e). These data revealed that PARP3 and G9a inversely co-regulate the expression of *Hif-2α*, *Mlh1* and *Runx3*. To examine whether PARP3 moderates G9a activity onto H3K9me2, we then compared the enrichment of this histone mark onto the promoters of the three genes in the PARP3^−/−3^ and PARP3^−/−18^ LN229 cells compared to the parental WT LN229 cells in normoxia and upon 1% O_2_ hypoxia (Sup. Fig. 4f–h). Surprisingly we found no significant difference in the establishment of H3K9me2 on the promoter of the genes in the PARP3-deficient cells compared to the parental WT cells cultured under normoxia or hypoxia. These results indicate another level of cooperation between both proteins for the regulation of these genes.

**Figure 6:**
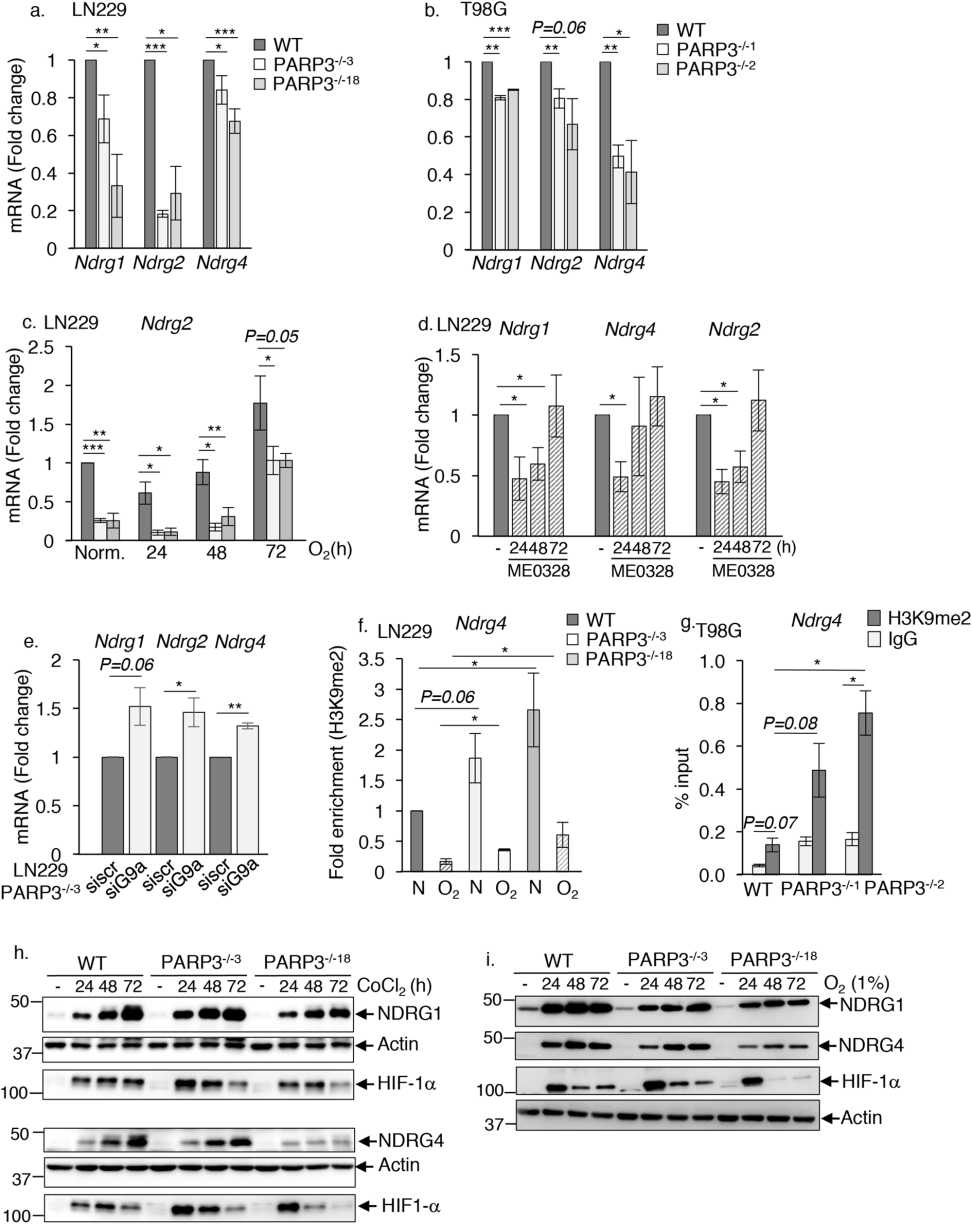
PARP3 moderates the G9a-mediated repression of the hypoxia responsive targets *Ndrg1, Ndrg2* and *Ndrg4.* (**a–c**) RT-qPCR analysis of *Ndrg1*, *Ndrg2* and *Ndrg4* in the parental WT and two PARP3-deficient clones of LN229 and T98G cells grown under normal conditions or under 1% O_2_ for the indicated time points. Data are expressed relative to *Actin*. Values represent means ± s.e.m. of 3 independent experiments and 3 technical replicates. *p < 0.05, **p < 0.01, ***p < 0.001. (**d**) ME0328-mediated chemical inhibition of PARP3 reproduces the selective downregulation of *Ndrg1, Ndrg2* and *Ndrg4* in normoxia. RT-qPCR analysis of *Ndrg1, Ndrg2* and *Ndrg4* in LN229 grown in normoxia in the absence or in the presence of ME0328 for the indicated time points. Data are expressed relative to *Actin*. Values represent means ± s.e.m. of 3 independent experiments and 3 technical replicates. *p < 0.05. (**e**) Recovery of expression of *Ndrg1*, *Ndrg2* and *Ndrg4* in the PARP3-deficient cells upon G9a silencing. The PARP3^−/−3^ LN229 cells were transfected with either sicontrol or siG9a for 72 h. The transcript levels of *Ndrg1, Ndrg2* and *Ndrg4* were analysed by RT-qPCR. Data are expressed relative to *Actin*. Values represent means ± s.e.m. of 3 independent experiments and 3 technical replicates. *p < 0.05, **p < 0.01. (**f**) Increased enrichment of H3K9me2 on the promoter of *Ndrg4* in PARP3-deficient LN229. H3K9me2 binding was assessed on the promoter region of *Ndrg4* by ChIP-qPCR in the parental WT versus the PARP3^−/−3^ and PARP3^−/−18^ LN229 cells grown in normoxia (N) or exposed to 1% O_2_ for 72 h. Enrichments were normalized to an IgG antibody used as ChIP negative control and fold enrichments were calculated relative to the parental LN229 grown in normoxia. Data are represented a percent of input and are means (± s.e.m) of three biological replicates. *p < 0.05. (**g**) Increased enrichment of H3K9me2 on the promoter of *Ndrg4* in PARP3-deficient T98G cells. H3K9me2 binding was assessed on the promoter region of *Ndrg4* by ChIP-qPCR in the parental WT versus the two PARP3^−/−1^ and PARP3^−/−2^ T98G cells. IgG were used as ChIP negative controls. Data are represented as percent of input and are means (± s.e.m) of three technical replicates of a representative experiment (n = 3) *p < 0.05. (**h**, **i**) The absence of PARP3 limits the hypoxia-induced upregulation of NDRG1 and NDRG4. The parental WT, PARP3^−/−3^ and PARP3^−/−18^ LN229 cells were maintained under CoCl2 (**h**) or 1% O_2_ (**i**) for the indicated time points. NDRG1, NDRG4, HIF-1α and actin were analysed by western blotting using the appropriate antibodies. Uncropped Western-blots are shown in the supplementary information file.

Considering that G9a/GLP1 complex was also found to inhibit HIF-1α transcriptional activity^27^, we next examined the expression of a subset of HIF-1α downstream target genes including *Glut1*, *Phd2*, *Phd3*, *Ndrg1, Ndrg2* and *Ndrg4*. (Fig. 6a–e and Sup. Fig. 5). *Ndrg* genes were selected because of their dysregulation following the chemical inhibition of G9a^30^, which we confirmed in the LN229 cell model (Sup. Fig. 5a). The expression of *Glut1*, *Phd2*, and *Phd3* was not impaired by the absence of PARP3 (Sup. Fig. 5b–d). In contrast, we found a selective down-regulation of the HIF target genes *Ndrg1, Ndrg2* and *Ndrg4* in both PARP3-deficient LN229 and T98G cell models and upon selective ME0328-mediated PARP3 inhibition in LN229 in normoxia (Fig. 6a–d). Under hypoxia, repression of *Ndrg2* was maintained in both PARP3-deficient LN229 cell lines, repression of *Ndrg1* was identified in the PARP3^−/−18^ cells while a transient repression of *Ndrg4* was detected in the PARP3^−/−3^ cells. (Fig. 6c, and Sup. Fig. 5e,f). We then measured the expression of the *Ndrg* genes in the PARP3^−/−3^ LN229 cells cultured in normoxia upon G9a silencing and found that *Ndrg1*, *Ndrg2* and *Ndrg4* were no longer repressed (Fig. 6e). In contrast, their expression was markedly increased in the siG9a versus the sicontrol PARP3^−/−3^ LN229 confirming that PARP3 and G9a inversely co-regulate the expression of all three *Ndrg1*, *Ndrg2* and *Ndrg4.* We next analysed the enrichment of H3K9me2 onto the promoters of these genes in the PARP3^−/−3^ and PARP3^−/−18^ LN229 cells compared to the parental WT LN229 cells in normoxia and upon 1% O_2_ hypoxia. We were not able to detect an enrichment of H3K9me2 onto *Ndrg1* in our cells*.* We did not detect more H3K9me2 onto *Ndrg2* in the PARP3-deficient cells compared to the parental WT cells cultured under normoxia or hypoxia suggesting that for this gene, G9a/PARP3 cooperation does not occur onto H3K9me2 (Sup. Fig. 5g). In contrast, we detected a significant increase in the establishment of H3K9me2 on the promoter of *Ndrg4* in the absence of PARP3 in LN229 that we validated further in the PARP3-deficient T98G cells (Fig. 6f,g). This increase was maintained under hypoxia in LN229 cells. Importantly, the enrichment of H3K9me2 on *Ndrg4* was significantly reduced in the PARP3^−/−2^ T98G cells upon G9a silencing, while a moderate though no significant reduction was detected in the PARP3^−/−3^ LN229 cells (Sup. Fig. 6). Moreover, we detected a weak increase in the enrichment of G9a on *Ndrg4* in the PARP3^−/−3^ LN229 cells (Sup. Fig. 7).

Together these data revealed that PARP3 tempers G9a-catalysed H3K9me2 specifically onto *Ndrg4* in both LN229 and T98G cells.

To validate the importance of PARP3, we also followed the protein expression levels of NDRG1 and NDRG4 upon CoCl_2_ or 1% O_2_-induced hypoxia in the WT versus the PARP3- deficient cell lines (Fig. 6h,i and Sup. Fig. 8a–d). Hypoxic conditions induced a significant increase in NDRG1 and NDRG4 protein levels. This increase was a little less important in the PARP3^−/−3^ LN229 cells and reduced in the PARP3^−/−18^ LN229 cells.

Overall, these results clearly reveal that PARP3 also moderates G9a activity onto a subset of hypoxia-responsive genes to help their expression. However, only for *Ndrg4*, this regulation takes place onto H3K9me2.

### PARP3 mediates the stability of cytoplasmic microtubules under hypoxia

**Figure 7:**
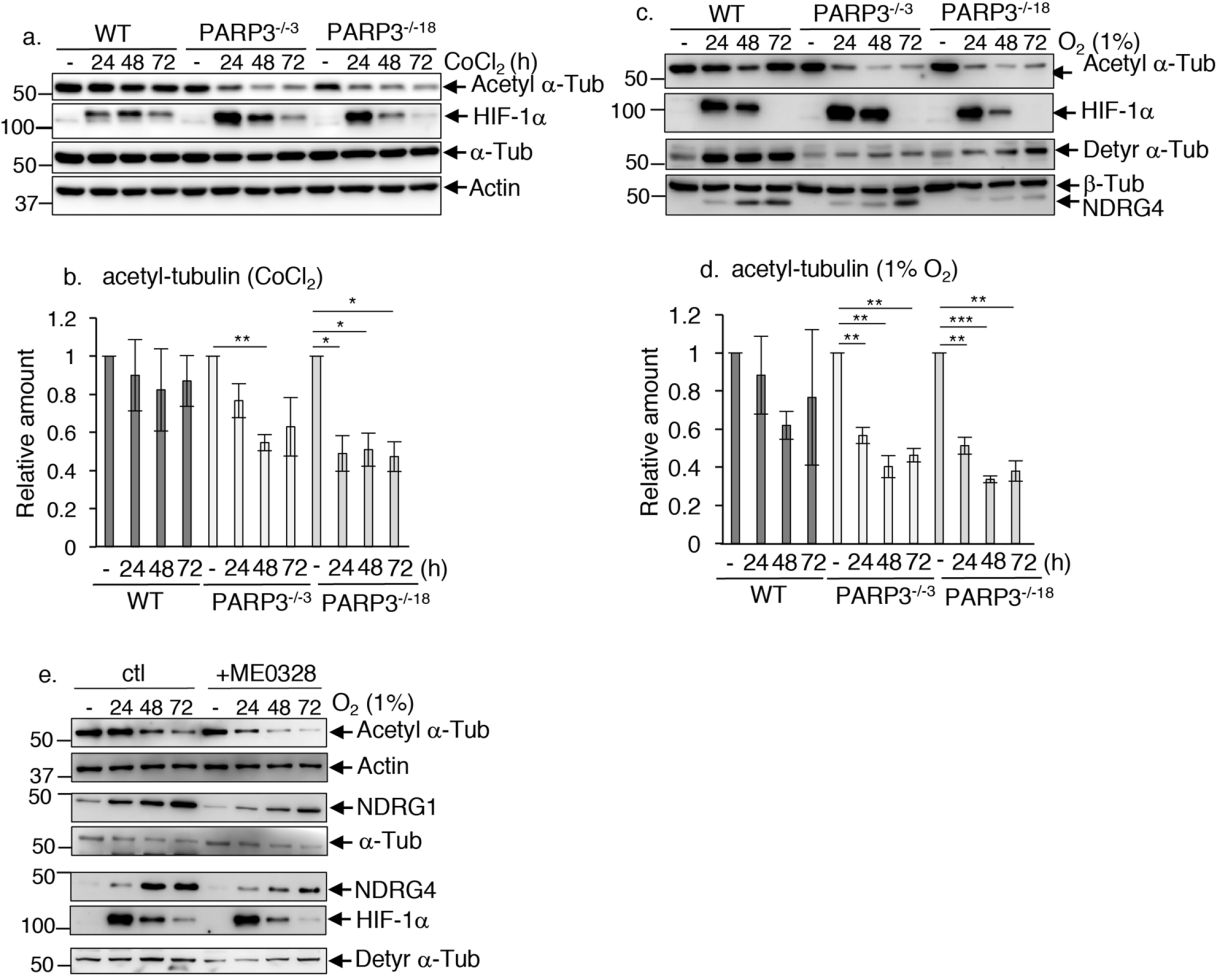
PARP3 deficiency destabilizes cytoskeleton microtubules under hypoxia. (**a**,**b**) PARP3 deficiency reduces tubulin acetylation during CoCl_2_-induced hypoxia. (**a**) The parental WT, PARP3^−/−3^ and PARP3^−/−18^ LN229 cells were maintained under CoCl_2_-induced hypoxia for the indicated time points. Acetyl α-Tubulin, HIF-1α α-tubulin and actin were analysed by western blotting using the appropriate antibodies. Uncropped Western-blots are shown in the supplementary information file. (**b**) Bar graphs depict the relative signal intensities of acetyl α-Tubulin versus α-Tubulin measured in three independent experiments using Image J. Mean values ± s.d are indicated. *p < 0.05, **p < 0.01. (**c**,**d**) PARP3 deficiency reduces tubulin acetylation under 1% O_2_-induced hypoxia. (**c**) The parental WT, PARP3^−/−3^ and PARP3^−/−18^ LN229 cells were cultured under 1% O_2_-induced hypoxia for the indicated time points. Acetyl α-Tubulin, detyr α-Tubulin, β-Tubulin, HIF-1α were analysed by western blotting using the appropriate antibodies. Uncropped Western-blots are shown in the supplementary information file. (**d**) Bar graphs depict the relative signal intensities of acetyl α-Tubulin versus β-Tubulin measured in three independent experiments using Image J. Mean values ± s.d are indicated. **p < 0.01, ***p < 0.001. (**e**) The chemical inhibition of PARP3 (ME0328) reduces tubulin acetylation, tubulin detyrosination, and impairs NDRG1 and NDRG4 upregulation under O_2_-induced hypoxia. Mock-treated and ME0328-treated LN229 cells were cultured under 1%O_2_-induced hypoxia for the indicated time points. Acetyl α-Tubulin, α-Tubulin, detyr α-Tubulin, HIF-1α, NDRG1, NDRG4 and actin were analysed by western blotting using the appropriate antibodies. Uncropped Western-blots are shown in the supplementary information file.

It is well-established that cell response to hypoxia involves a significant reorganization of the cytoskeleton during which the strict regulation of microtubules stability and dynamics plays a prime role. Among the post-translational modifications that control microtubule properties, α-tubulin acetylation has been defined as a marker of stable, polymerized microtubules with a protective role from mechanical breakage^36^. Stress-induced decrease of tubulin acetylation reveals fragility of the microtubule network. In mammals and plants, NDRG proteins have been linked with microtubule acetylation and stability^37,38^. These findings together with previous data demonstrating that PARP3 regulates the stability of the mitotic spindle prompted us to investigate the contribution of PARP3 in cytoskeleton rearrangement under hypoxia^3^. To this end, we analysed the levels of acetylated α-tubulin in our WT and PARP3^−/−^ LN229 cell lines upon CoCl_2_ treatment or an exposure to 1% O_2_ (Fig. 7a–d). In both experimental conditions, we detected a hypoxia-induced decrease in the levels of acetylated α-tubulin in the WT cells as expected. This decrease was significantly enhanced in the PARP3^−/−3^ and PARP3^−/−18^ LN229 cells indicating serious microtubules instability. Consistently, in the condition of 1% O_2_, we also observed reduced tubulin detyrosination, another marker of microtubule stability, in the PARP3^−/−3^ and PARP3^−/−18^ cells compared to the WT cells. In support of a role of PARP3 catalytic activity, reduced α-tubulin acetylation, reduced α-tubulin detyrosination, and impaired upregulation of NDRG1 and NDRG4 were also detected upon the ME0328-mediated chemical inhibition of PARP3 activity (Fig. 7e). Moreover, although following a different profile and at a lower extend, impaired α-tubulin acetylation under hypoxia was also reproducibly detected in the two PARP3-deficient T98G cells compared to their WT counterpart (Sup. Fig. 9a).

### The absence of PARP3 increases the sensitivity of LN229 cells to microtubule-destabilizing agents

**Figure 8:**
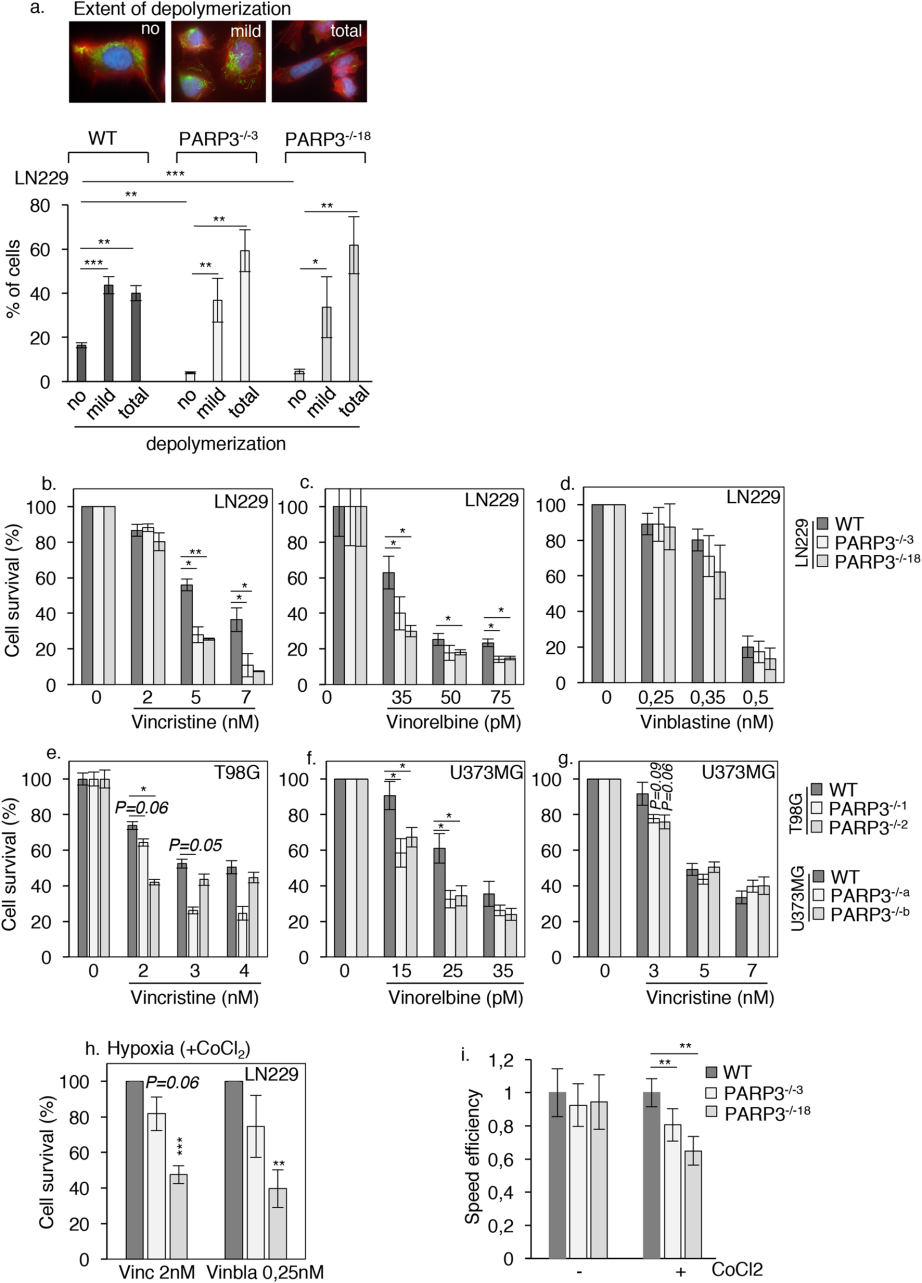
Microtubules instability in PARP3-deficient cells impairs motility and induces sensitivity to nocodazole and vinca-alkaloids. (**a**) PARP3-deficient cells are more sensitive to nocodazole-induced microtubules depolymerization. The parental WT and the PARP3^−/−3^ and PARP3^−/−18^ LN229 cells were treated with nocodazole and immunostained for acetylated α-Tubulin (green). Actin was labeled using Alexa 594-phalloidin (red). The nucleus was stained with Dapi. The number of cells showing total, mild or no depolymerization was counted. Values represent the mean + /- s.d of 3 independent experiments and a total counting of > 350 figures per experiment. *p < 0,05, **p < 0,01, ***p < 0,001. (**b-g**) The absence of PARP3 sensitizes glioblastoma cells to microtubule-destabilizing agents. Dose response clonogenic survival curves of the wild type (WT) and two PARP3-deficient clones of LN229, T98G or U373MG cell lines treated with vincristine, vinorelbine or vinblastine and grown under normoxia. Results represent the mean values of three independent experiments ± s.d. For (**c**) and (**e**), results represent the mean values of three technical replicates ± s.d. of a representative experiment. *p < 0.05, **p < 0.01. (**h**) Hypoxia exacerbates the sensitivity of PARP3-deficient LN229 cells to vincristine and reveals sensitivity to vinblastine. The parental WT and the PARP3^−/−3^ and PARP3^−/−18^ LN229 cells were cultured under CoCl_2_-induced hypoxia and treated with non-toxic doses of vincristine and vinblastine. Results represent the mean values of three independent experiments ± s.d. **p < 0.01,***p < 0.001. (**i**) PARP3 deficiency alters cell motility (scratch wound closure) under hypoxic stress. The parental (WT) and the PARP3^−/−3^ and PARP3^−/−18^ LN229 cells were either left untreated (−) or exposed to CoCl_2_ for 48 h (+) before performing a scratch wound assay. CoCl_2_ was maintained during the wound healing. Histogram depicts the speed efficiency relative to the WT set to 1 and calculated from analysis of video recording of the in vitro scratch wounds. Error bars represent the means of 3 independent experiments ± s.d. **p < 0.01.

To further confirm microtubules fragility in the absence of PARP3, we then compared the efficiency of nocodazole to depolymerize microtubules in the WT and the PARP3-deficient LN229 cells by analyzing the pattern of acetylated tubulin by immunofluorescence staining (Fig. 8a). In interphase, untreated cells display a homogeneous pattern of long stretches of clustered microtubules extending outward from the centrosome toward the periphery of the cell. No difference was observed between the WT and the PARP3-deficient cells. A short mild treatment of WT cells with nocodazole induced the accumulation of cells with either short stretches of depolymerized microtubules randomly distributed in the cell or cells with no remaining tubulin staining reflecting complete depolymerization. We analysed the distribution of these figures in the nocodazole-treated PARP3^−/−3^ and PARP3^−/−18^ cells versus the parental WT cells. Consistent with microtubules weakness, we detected a significant decrease in the percentage of cells with polymerized long stretches of tubulins compared to the WT cells and an increase in the percentage of cells with complete depolymerized microtubules. These results confirmed that the absence of PARP3 reduces the stability of cytoskeletal microtubules in normoxia.

Following this result, we hypothesized that the absence of PARP3 may affect the sensitivity of glioblastoma cells to microtubule-destabilizing agents. To address this hypothesis, we treated our LN229 and T98G cell lines with vincristine, vinblastine and/or vinorelbine in normoxia and analysed cell viability using clonogenic assays. We also engineered PARP3-deficient U373MG cell lines (Sup. Fig. 9b). When grown in normoxia, PARP3-deficient LN229 were highly susceptible to the cytotoxic effect of vincristine and vinorelbine but not vinblastine, PARP3-deficient T98G cells were sensitive to vincristine and PARP3-deficient U373MG were sensitive to vinorelbine, and at the lowest concentration a moderate sensitivity to vincristine was consistently observed (Fig. 8b–g). Sensitivity to vincristine was rescued in the PARP3^−/−3^ LN229 by the re-expression of Flag-PARP3^WT^ but also Flag-PARP3^HE^ demonstrating the importance of PARP3 protein beyond its catalytic activity (Sup. Fig. 9c). Moreover, the exposure of LN229 cells to vincristine and vinblastine at doses that were non-toxic in normoxia, revealed a sensitivity of the PARP3-deficient LN229 to vinblastine, and enhanced their sensitivity to vincristine when cultured under CoCl_2_-induced hypoxia (Fig. 8h).

Given the importance of microtubules acetylation but also Nfasc and Parvb in cell motility, we also examined the effect of PARP3 deficiency on cell motility in normoxia and under CoCl_2_-induced hypoxia using a wound healing assay (Fig. 8i and Sup. Fig. 9d). While the absence of PARP3 had no impact on the speed efficiency of LN229 cells when grown in normal culture conditions, under CoCl_2_, the motility of the PARP3^−/−3^ and PARP3^−/−18^ cell lines was reduced compared to the control cells suggesting that hypoxia-induced microtubules weakness impairs migration of the PARP3-deficient LN229 cells.

Collectively, these data suggest that PARP3 is required to regulate the stability of cytoskeletal microtubules under hypoxia and its absence increases the susceptibility of glioblastoma cells to microtubule destabilizing agents.

## Discussion

In this study we identified PARP3 as a novel binding partner of G9a. By exploring the functional aspects of this association in the model of glioblastoma, we found that PARP3 and its catalytic activity helps to restrain G9a-mediated repression of the adhesion genes *Nfasc* and *Parvb* and the hypoxia-regulated genes *Hif-2α*, *Mlh1, Runx3, Ndrg1, Ndrg2* and *Ndrg4*. Intriguingly, PARP3 controls G9a-catalyzed H3K9me2 at the gene specific level onto *Nfasc*, *Parvb* and *Ndrg4* specifically, suggesting an other level of cooperation for the silencing of the hypoxia genes *Hif-2α*, *Mlh1*, *Runx3*, *Ndrg1* and *Ndrg2*. Because G9a acts predominantly in an heterodimeric complex with GLP1 and that this complex appears to be the functional H3K9 methyltransferase in vivo^32^, we verified that the G9a/GLP1 heterodimer formation was maintained in the PARP3-deficient cells. Also the interaction of Wiz with this complex, a core subunit needed for G9a stability, H3K9 methylation activity and gene repression, was conserved in the absence of PARP3^17,19^. These results suggest that PARP3 might not be necessary for the formation of the G9a/GLP1/Wiz complex. A previous study has demonstrated the existence of a significant interaction between G9a and the Polycomb Repressive Complex 2 (PRC2)^21,22^. In vivo, this association was described to secure the epigenetic silencing of a subset of developmental and neuronal genes in mESCs. Mechanistically, G9a enzymatic activity was found to control PRC2 recruitment and H3K27 trimethylation at these specific genomic regions. Interestingly, co-immunoprecipitation experiments identified an association of PARP3 with several core components of the PRC2 complex including the catalytic subunit EZH2, Suz12 and YY1^10^. Thus, it is tempting to propose that through its association with G9a and by modulating its activity, PARP3 contributes to fine-tune PRC2-mediated trimethylation of H3K27. However, total levels of H3K27me3 were not impaired by the absence of PARP3 nor its chemical inhibition (data not shown) suggesting that PARP3 does not control the G9a-PRC2 axis at the global level of H3K9 trimethylation. That this occurs at specific genomic regions such as the common targets *Hif-2α*, *Mlh1*, *Runx3*, *Ndrg1*, *Ndrg2* remains an issue.

Since PARP3 and G9a control the expression of common hypoxia-associated genes, we speculated on a possible functional cooperation between both enzymes in cell response to hypoxia, a hallmark of glioblastoma invasion. This hypothesis is supported by earlier findings reporting that G9a plays as an epigenetic mediator of hypoxia signaling^28–30^ and that PARP3 mediates a hypoxic response in mouse neural stem progenitor cells after acute hypoxia-ischemia^35^. In agreement with this latter result, we show here that PARP3 is implicated in the regulation of hypoxia signaling pathways in glioblastoma cells. *Parp3* expression is increased upon 1% O_2_-induced hypoxia and its absence in LN229 confers high vulnerability to chronic hypoxia. Our data reveal that the PARP3-G9a cooperation for the transcriptional regulation of *Hif-2α*, *Mlh1*, *Runx3*, *Ndrg1*, *Ndrg2* and *Ndrg4* occurs both in normoxia and under hypoxia stress.

Mounting evidences have associated the hypoxic response with dramatic cytoskeleton remodeling. It involves the reorganization of microtubules determined by a decrease in the acetylation and detyrosination of α-tubulin, both of which are markers of microtubules stability. In a previous study, we have identified a key contribution of PARP3 in the assembly and stability of the mitotic spindle microtubules^3^. Moreover, NDRG proteins have been suggested to promote tubulin acetylation and stability^38^. Thus to further progress with the role of PARP3 in hypoxic stress response, we made the hypothesis that PARP3 could regulate the acetylation and stability of cytoskeletal microtubules. Our data reveal an accelerated hypoxic stress-induced decrease in α-tubulin acetylation and detyrosination in the PARP3 knockout glioblastoma cells and upon PARP3 inhibition, indicating weakened cytoskeleton microtubules. These findings suggest that PARP3 protects from hypoxia-induced disassembly of cytoskeletal microtubules. To which extend and how PARP3-mediated regulation of G9a contributes to α-tubulin acetylation and integrity remains a relevant question to explore in future studies. That PARP3 supervises G9a-mediated repression of *Ndrg1, Ndrg2* and *Ndrg4* and that its absence impairs the hypoxia-induced increase in NDRG1 and NDRG4 levels led us to postulate on the existence of a PARP3-G9a-NDRG axis that at least partly mediates cell response to hypoxia. In line with this, NDRG proteins have previously been linked with α-tubulin stability^38^. Yet, clonogenic assays revealed a weak potentializing effect of NDRG4 depletion on the sensitivity of PARP3-deficient LN229 cells to vincristine thus suggesting an additional level of regulation by the NDRG proteins (Sup. Fig. 10). The PARP3-G9a axis might also implicate other yet unidentified G9a-regulated genes, a G9a-mediated lysine methylation of an unknown protein key for tubulin stability, or simply the G9a-catalysed methylation of α-tubulin. This latter hypothesis is supported by recent findings describing the emergence of dual-function methyltransferases that methylate both histones and tubulin among them SETD2^39^ or SMYD2^40^. Comparative G9a CHIPseq experiments combined with comparative mass spectrometry-based proteomics to explore G9a partners and targets performed in the WT and PARP3-deficient cell models will help to scrutinize these hypotheses in future studies.

Given that PARP3-deficient cells displayed microtubules instability, we predicted vulnerability to microtubule-destabilizing agents. Consistently, we identified a context-specific hypersensitivity of three different PARP3-deficient cell lines LN229, T98G and U373MG to either vincristine, vinblastine or vinorelbine. The absence of PARP3 also sensitized LN229 to nocodazole revealed by increased cytoskeletal microtubules depolymerization. Moreover, vulnerability to vincristine and vinblastine were further enhanced under hypoxic stress in PARP3-deficient LN229 cells consistent with the contribution of PARP3 to maintain microtubules stability in hypoxic conditions.

Acetylation of α-tubulin was also described to promote cell migration^41^. Accordingly, PARP3 knockout LN229 displayed impaired reduced migration ability when grown under hypoxia further supporting the role of PARP3 in microtubules stability upon hypoxic stress.

In conclusion, this work identified PARP3 as a novel interactor and regulator of G9a. PARP3 ADP-ribosylates G9a in vitro and helps to adjust G9a-mediated transcriptional inhibition of the *Nfasc* and *Parvb* genes involved in cell adhesion and of the *Hif-2α*, *Mlh1, Runx3, Ndrg1, Ndrg2* and *Ndrg4* genes that promote cell response to hypoxia. For a subset of these genes, PARP3 controls G9a for the enrichment of the repressive mark H3K9me2.

In addition, despite an absence of overt phenotype in the PARP3-knockout glioblastoma cells exemplified by normal proliferation and normal in vivo tumor progression, we provide strong evidence that PARP3 dysfunction participates in enhancing the fragility of cytoskeletal microtubules that confers vulnerability to microtubule-destabilizing agents. We speculate that this event is at least partly mediated by a PARP3-G9a crosstalk that needs to be specified. In the future, these findings will inspire novel therapeutics strategies for the use of PARP3-specific inhibitors combined with microtubules targeting agents to overcome tumor hypoxia as a barrier to effective cancer treatment in glioblastoma.

## Materials and methods

### Reagents and plasmids

Vincristine and CoCl_2_ were purchased from Sigma-Aldrich. Vinblastine was purchased from Enzo Life sciences. Vinorelbine was purchased from Tocris. The PARP3 inhibitor ME0328 has been described^42^. The EGFP-G9a construct has been purchased from Addgene (33025). The pMXPIE plasmids encoding either Flag, the catalytically active Flag-PARP3^WT^ or the dead mutant Flag-PARP3^HE^ have been described^6^.

### Cell culture, cell proliferation and hypoxia treatment

All cells were a generous gift from M. Dontenwill (Laboratoire de Bioimagerie et pathologies, Illkirch). LN229, U373MG, SF763, LN18, T98G, U87MG, LNZ308 and SF767 were maintained in MEM medium supplemented with 10% FCS and 1% gentamicin. LN443 and LN444 were maintained in DMEM-1 g/L d-glucose medium supplemented with 10% FCS and 1% gentamicin. All cell lines were maintained at 37 °C in a humidified 5% CO2 atmosphere.

To measure cell growth, cells were seeded into 6-well plates (500.000 cells/well) in triplicates and counted daily for 4 days.

Gene specific siRNA for G9a (ON_TARGET plus human G9a L-006937-00) and negative control siRNA (scr) were obtained from Horizon Discovery. Cells were transfected with 25 nM siRNA using JetPrime (PolyPlus Transfection) according to the manufacturer’s instructions and processed 72 h later.

Hypoxia was induced either chemically by growing cells in medium supplemented with 100 μM of the hypoxia mimetic agent CoCl_2_ for the indicated time points, or by growing cells in low oxygen conditions using a tri-gas incubator flushed with a gas mixture containing 1% O_2_, 5% CO_2_ and balanced with N_2_. When analyzing the impact of ME0328, cells were pre-treated with the compound for 24 h before hypoxia and the compound was maintained throughout the experiment.

### Knockout of PARP3 using CRISPR/nCas9-mediated genome editing

CRISPR/Cas9 knockout cells were generated by co-transfection of the cells with two plasmids either co-expressing 2 sgRNAs targeting exon 2 and nCas9-EGFP, or co-expressing 2 sgRNAs targeting exon 5 and nCas9-mCherry. The sgRNAs sequences are as following: Exon 2: gRNA1: GCCTCAGCGGTGGAGCGGAA, gRNA2: AGAGAAGCGCATAATCCGCG, Exon 5: gRNA3: GTTAGTGATGAGCTTCTGCG, gRNA4: CACCATGGCCCTCATGGACC. LN229 cells were transfected using FuGENE^6^ (Promega). T98G and U373MG cells were transfected using JetPrime according to the manufacturer’s instructions. Seventy two hours after transfection, GFP+/mCherry+ cells were sorted by flow cytometry and single cells were plated in 96-wells plates. Singles colonies were picked, amplified and verified by sequencing and western blotting for the absence of PARP3. For each cell line, two PARP3 knockout clones were selected.

### Generation of PARP3-rescued cell lines

PARP3^−/−3^-LN229 cells were transfected with 10 µg of pMXPIE plasmid encoding either Flag, the catalytically active Flag-PARP3^WT^ or the dead mutant Flag-PARP3^HE^ using FuGENE^6^. Three days after transfection, the cells were sorted for EGFP expression by flow cytometry and amplified. The expression of Flag-PARP3^WT^ and Flag-PARP3^HE^ was verified by western blotting.

### Cell extracts, immunoprecipitation and western blotting

Whole cell extracts were prepared as described previously^6^. Cells were lysed in cold RIPA buffer (50 mM TrisHCl pH 8, 1% Triton X-100, 0.25% Sodium deoxycholate, 150 mM NaCl, 1 mM EDTA, 50 mM NaF, 20 mM Sodium pyrophosphate, 1 mM Sodium orthovanodate, 1 mM Pefabloc (Roche) and protease inhibitor cocktail «complete mini EDTA free» (Roche)) for 10 min on ice. After centrifugation at 13,000 rpm for 15 min at 4 °C, clear lysates were quantified using the Bradford assay (BioRad). For nuclear extracts, cells were resuspended in hypotonic buffer (10 mM TrisHCl pH 7.3, 10 mM KCl, 1.5 mM MgCl_2_, 10 mM β-mercaptoethanol, 0.2 mM PMSF) and homogenised on ice by using a glass Dounce homogenizer (pestle B). After centrifugation at 1600 rpm for 5 min at 4 °C, nuclear pellets were resuspended in cold extraction buffer (15 mM TrisHCl pH 7.3, 0,4 M NaCl, 1 mM MgCl_2_, 1 mM EDTA, 10% glycerol, 10 mM β-mercaptoethanol, 0.2 mM PMSF), incubated 30 min on ice and centrifuged at 13,000 rpm for 30 min at 4 °C. The supernatant was used as the nuclear extract fraction. For all extracts, equivalent amounts of proteins were analysed by 10% SDS-PAGE and immunoblotting using the appropriate antibodies (Sup. Table 1). Protein bands were visualized using ECL-PLUS detection system (Amersham biosciences) and the images were capture using the Image Quant LAS500 imaging system (GE Healthcare Life Science). Signal intensities were analysed using ImageJ.

For immunoprecipitation experiments, whole cell RIPA lysates were first treated with DNAse I (Promega) (3U DNAse I, 6 mM MgCl_2_, 10 mM CaCl2) for 3 min at 25 °C followed by 45 min on ice. Then lysates were diluted 4X in dilution buffer (15 mM TrisHCl pH 8, 150 mM NaCl) and incubated 4 h at 4 °C in the presence of agarose beads coupled to Flag antibody (anti-Flag M2 Affinity gel A2220, Sigma) or agarose beads coupled to GFP (Chromotek). Beads were washed twice with washing buffer 1 (15 mM TrisHCl pH 8, 250 mM NaCl) and twice with washing buffer 2 (15 mM TrisHCl pH 8, 150 mM NaCl). Beads were then resuspended in Laemli buffer and subjected to 10% SDS-PAGE and immunoblotting using the appropriate antibodies (Sup. Table 1). Protein bands were visualized using ECL-PLUS detection system (Amersham biosciences) and the images were captured using the Image Quant LAS500 imaging system (GE Healthcare Life Science). When indicated, cells were transfected with EGFP or EGFP-G9a 72 h before processing for IP as detailed above.

### Indirect immunofluorescence microscopy

Cells were grown on glass coverslips (2 × 10^5^ cells/6-well plates) for 24 h and processed for immunofluorescence as described^3^. Briefly, cells were washed twice in PBS 1X, fixed for 15 min at room temperature in 4% formaldehyde diluted in PBS 1X and washed three times for 10 min in blocking buffer (PBS 1X/0.1% Triton X-100/0.1% skimmed milk). Cells were then incubated overnight at 4 °C with the anti-acetylated α-Tubulin antibody diluted in blocking buffer, washed three times for 10 min with blocking buffer and incubated for 2 h at room temperature in Alexa Fluor conjugated secondary antibody followed by a 30 min incubation with Alexa Fluor-568 Phalloidin. Slides were mounted with the Fluoromount-G mounting medium containing DAPI. Images were captured using a Leica TCS40D microscope (Leica Microsystems GmbH) equipped with an ORCA-ER chilled CCD camera (Hammamatsu) and the capture software Openlab (Improvision).

### Measurement of scratch-induced migration

For wound-healing assays, cells were seeded in triplicates in 6-well plates and cultured with or without 100 µM CoCl_2_ until forming a monolayer. A linear wound was created using a 200 µL sterile pipette tip. Wound healing was monitored by videomicroscopy with a wide field Leica DMIRE 2 microscope placed in an incubation chamber maintained at 37 °C and 5% CO_2_ and in an humid atmosphere. The microscope was equipped with a photometrics Prime sCMOS camera and the Imaging capture software Metamorph. Images of the cells were captured every 20 min for 24 h using a 10 × phase contrast lens. The cell migration capacity was analyzed with Image J software.

### RNA sequencing and qRT-PCR

RNA sequencing was performed as previously described^35^. Total RNA was extracted using the RNeasy Mini-Kit (Qiagen) following the manufacturer’s instructions.

For RNAseq, total RNA-Seq libraries were generated from 1000 ng of total RNA using TruSeq Stranded Total RNA LT Sample Prep Kit with Ribo-Zero Gold (Illumina, San Diego, CA), according to manufacturer's instructions. Briefly, cytoplasmic and mitochondrial ribosomal RNA (rRNA) were removed using biotinylated, target-specific oligos combined with Ribo-Zero rRNA removal beads. Following purification, the depleted RNA was fragmented into small pieces using divalent cations at 94 °C for 2 min. Cleaved RNA fragments were then copied into first strand cDNA using reverse transcriptase and random primers followed by second strand cDNA synthesis using DNA Polymerase I and RNase H. Strand specificity was achieved by replacing dTTP with dUTP during second strand synthesis. The double stranded cDNA fragments were blunted using T4 DNA polymerase, Klenow DNA polymerase and T4 PNK. A single 'A' nucleotide was added to the 3' ends of the blunt DNA fragments using a Klenow fragment (3′ to 5′exo minus) enzyme. The cDNA fragments were ligated to double stranded adapters using T4 DNA Ligase. The ligated products were enriched by PCR amplification (30 s at 98 °C; [10 s at 98 °C, 30 s at 60 °C, 30 s at 72 °C] × 12 cycles; 5 min at 72 °C). Surplus PCR primers were further removed by purification using AMPure XP beads (Beckman-Coulter, Villepinte, France) and the final cDNA libraries were checked for quality and quantified using capillary electrophoresis. Sequencing was performed on an Illumina HiSeq 4000 in a 1 × 50 bp single end format. Reads were preprocessed using cutadapt 1.10 in order to remove adaptors and low-quality sequences and reads shorter than 40 bp were removed for further analysis. Remaining reads were mapped to Homo sapiens rRNA and ERCC Spike-In sequences using bowtie 2.2.8^43^ and reads mapped to those sequences were removed for further analysis. Remaining reads were aligned to hg38 assembly of Homo sapiens with STAR 2.5.3a^44^. Gene quantification was performed with htseq-count 0.6.1p1^45^, using “union” mode and Ensembl 91 annotations. Differential gene expression analysis was performed using DESeq2 1.16.1^46^. Bioconductor R package on previously obtained counts (with default options). P-values were adjusted for multiple testing using the Benjamini and Hochberg method^47^.

RT-qPCR was performed essentially as described^35^. Briefly, 1 μg of DNase-treated RNA was reverse transcribed using the Maxima Reverse Transcriptase (Thermofisher Scientific) according to the manufacturer’s instructions. Real-time PCR was performed using the SYBR™ Green PCR Master Mix kit following the manufacturer’s instructions (Applied biosystems) combined with the Applied Biosystems StepOne™ (Life technologies) detection system. For very low expressing genes (*Acan*, *Wnt10b*, *Lyn*, *Fbln5*), the Taqman™ Universal Master Mix II (Applied Biosystems) protocol was used. The PCR products were analysed with the StepOne Software. The quantity of PCR products was determined by the 2ΔΔ^−CT^ quantification method. All samples were analysed in triplicates and normalized using the *Gapdh* or *Actin* housekeeping genes as indicated. The primer sequences used for qPCR are listed in the Sup. Table 2.

### Chromatin immunoprecipitation (ChIP)

For each ChIP sample, four 150 mm-cell culture dishes at 80–90% confluence were used. DNA and proteins were cross-linked with 1% formaldehyde directly added in the culture medium for 10 min at room temperature under shaking. The reaction was stopped by adding 0,125 M of glycine and shaking during 5 min. Fixed cells were washed twice with ice-cold PBS 1 ×, and collected. To prepare chromatin, cells were resuspended in 3 mL ice-cold lysis buffer (0.5 M PIPES pH 8, 2 M KCl, 10% NP-40 and protease inhibitor cocktail «complete mini EDTA free» (Roche)) and incubated 10 min on ice. Cells were homogenized 40 times on ice with a dounce. Nuclei were then pelleted by centrifugation at 4000 rpm for 5 min at 4 °C, resuspended in 750 μL nuclear extraction buffer (1 M Tris pH 8.1, 5 M EDTA pH 8, 10% SDS and protease inhibitor cocktail) and lysed for 10 min on ice. Chromatin was then sonicated for 20 min using the covaris (20% duty cycle, 200 cycle per bust, 200 W) yealding genomic DNA fragments with a bulk size of 100 to 500 bp. Samples were diluted 5 × in dilution buffer (10% SDS, 10% Triton X-100, 0,5 M EDTA pH 8,1, 1 M Tris pH 8,1, 5 M NaCl and protease inhibitor cocktail) and pre-cleared with 200 μL protein A/G agarose beads for 2 h at 4 °C by rotary mixing. After centrifugation at 4000 rpm for 10 min at 4 °C, 100 μL of the supernatant was collected as input. An equivalent amount of 50 μg of extract was then immunoprecipitated using the specific antibodies (Sup. Table 1) overnight at 4 °C and immune complexes were recovered by adding 100 μL of pre-blocked agarose beads for an additional 2 h at 4 °C. Beads were washed once for 5 min with dialysis buffer (0.5 M EDTA, 1 M Tris pH 8.1, 20% N-Lauroylsarcosine sodium salt and protease inhibitor cocktail), and five times for 10 min with washing buffer (3 M Tris pH 8.1, 5 M LiCl, 10% NP-40, 10% sodium deoxycholate and protease inhibitor cocktail) and twice with Tris–EDTA pH8 (TE). Beads were finally resuspended in 200 μL TE. To reverse the crosslink, RNAse A (50 μg/mL) was added to the samples for 30 min at 37 °C under shaking (800 rpm) followed by 4 μL of 10% SDS and incubation overnight at 70 °C under shaking (1300 rpm). The bounded DNA was eluted from the beads by adding proteinase K (0.2 mg/mL) at 45 °C for 90 min under shaking (800 rpm). The immunoprecipitated DNA was purified by phenol–chloroform extraction and precipitated overnight with ethanol (100%, 2.5 V), sodium acetate (3 M, 0.1 V) and 1 μL glycogen at − 20 °C. The precipitated DNA was resuspended in water and analysed by real-time qPCR. Reactions were run in triplicates on the Applied Biosystems StepOne™ cycler by using the KAPA SYBR FAST qPCR polymerase kit following the manufacturer’s instructions (Clinisciences). The primers used for ChIP-qPCR are listed in Sup. Table 2. ChIP-qPCR results are represented as percentage of IP/input signal (% input).

### In vitro PARylation

To analyse PARP3-catalysed ADP-ribosylation of G9a, HEK 293 cells were transfected with either pEGFP-G9a or pEGFP as control for 72 h. Cleared RIPA whole cell extracts (1–1.5 mg) were diluted 4X in dilution buffer (15 mM TrisHCl pH 8, 150 mM NaCl) and incubated for 2 h at 4 °C in the presence of agarose beads coupled to GFP (Chromotek). After centrifugation at 5000 rpm at 4 °C for 5 min, beads were washed 4 times with washing buffer (15 mM TrisHCl pH 8, 250 mM NaCl). Next, beads were incubated for 1 h at 30 °C in 50 µL of reaction buffer containing biotinylated NAD^+^ (Trevigen ref. 4670-500-01, 2,5 μM), DNase I-treated calf thymus DNA (#ALX-840-040-C010, Enzo, 200 ng/μL), purified recombinant human PARP3 (Met2-hPARP3, 1.4 μg/μL^48^) and 5 μL 10X reaction buffer (50 mM TrisHCl pH8, 100 mM NaCl, 1 mM DTT). When indicated the PARP3 inhibitor ME0328 (20 µM) was added in the reaction buffer. The beads were then washed extensively in PBS 1X to reduce the auto-modified PARP3. Proteins were eluted by boiling in Laemmli buffer, separated on a 8–20% SDS-PAGE gel and transferred onto a nitrocellulose membrane. After blocking with PBS 1 ×-0,1% Tween-5% BSA, the membrane was incubated for one hour at room temperature with the Streptavidine-Alexa 680 (ThermoFisher S32358, diluted at 1:30,000 in PBS-0.01% Tween-0.5%BSA). Images were captured using an Odyssey infrared imaging system. Loaded EGFP fusion proteins were verified by immunoblotting using the appropriate antibody.

### In vivo tumorigenicity experiments

Experiments were performed in accordance with relevant guidelines and regulations. The study is reported in accordance with ARRIVE guidelines. Animal protocols were approved by the Ministry of Higher Education in Research and Innovation and the local ethics committee Cremeas (Comité Régional d'Ethique en Matière d'Expérimentation Animale de Strasbourg). Female athymic nude mice (S/SOPF SWISS NU/NU) were purchased from Charles Rivers Laboratories. Xenografts studies were performed as previously described^6^. Briefly, 5 × 10^6^ parental WT, PARP3^−/−3^-Flag, PARP3^−/−3^-Flag-PARP3^WT^, or PARP3^−/−3^-Flag-PARP3^HE^ LN229 cell lines in 50% of Matrigel (Corning) were implanted subcutaneously into both flanks of the 8-weeks-old nude mice under Isoflurane anesthesia. Tumor volumes were calculated from caliper measurements by length (L) and width (W) by using the formula: Tumor volume (V mm^3^) = length × (Width)^2^/2.

### Statistical analysis

Unless otherwise indicated, all experiments were performed as 3 technical replicates and at least 3 independent repeated experiments. Means, s.d and s.e.m were analysed using Microsoft Excel formula. Two-tailed unpaired Student’s t-test was used to compare the statistical difference between indicated groups. A P-value < 0.05 was considered statistically significant for all comparisons.

## Supplementary Information

Supplementary Information.

## Data Availability

The authors declare that all data supporting the findings in this study are available within the paper (and in Supplementary information). All data are available from the corresponding authors upon reasonable request. RNA seq raw data have been deposited in the Gene Expression Omnibus (GEO) under Accession Number GSE173801.
