# Supplementary material for: PARP3 supervises G9a-mediated repression of adhesion and hypoxia-responsive genes in glioblastoma cells
Source: Sci Rep. 2022 Sep 15;12:15534. doi: 10.1038/s41598-022-19525-6 (PMC9478127; doi:10.1038/s41598-022-19525-6)
Supplement: Supplementary file 1 — Supplementary Information. [file 41598_2022_19525_MOESM1_ESM.pdf]

SUPPLEMENTAL INFORMATION

PARP3 supervises G9a-mediated repression of adhesion and hypoxia-responsive genes in glioblastoma cells.

Nguekeu-Zebaze, L. *et al*.

Sup. Fig. 1: Sequencing analysis of the *PARP3*-deleted clones.
Sup. Fig. 2: PARP3 interacts with G9a.
Sup. Fig. 3: The chemical inhibition of G9a or its silencing reduces the enrichment of H3K9me2 on the promoter of *Nfasc and Parvb* in PARP3-deficient cells.
Sup. Fig. 4: PARP3 regulates the hypoxia-responsive genes *Hif-2α*, *Mlh1* and *Runx3* in common with G9a.
Sup. Fig. 5: PARP3 regulates the hypoxia-responsive genes *Ndrg1*, *Ndrg2*, *Ndrg4* in common with G9a.
Sup. Fig. 6: The depletion of G9a reduces the enrichment of H3K9me2 on the promoter of *Ndrg4* in PARP3-deficient T98G cells.
Sup. Fig. 7: Increased enrichment of G9a on the promoter of *Ndrg4, Nfasc and Parvb* in PARP3-deficient LN229 cells.
Sup. Fig. 8: The absence of PARP3 impairs efficient induction of NDRG1 and NDRG4 under hypoxia.
Sup. Fig. 9: The absence of PARP3 compromizes cell response to hypoxia.
Sup. Fig. 10: The depletion of NDRG4 weakly enhances the sensitivity of PARP3$^{-/-3}$ LN229 to vincristine.

Supplementary Table 1: List of antibodies used in the study.
Supplementary Table 2: List of primers used in the study.
Material and Methods: Reagents

Uncropped figures.
For all experiments (except 1a), uncropped blots are shown. Membrane were often cut to enable blotting with multiple antibodies. When necessary, for clearer results, the same biological replicate was loaded on different gels for the figure. For figure 1a, the different original exposure times available are shown.

LN229

PARP3$^{-/-3}$ (both alleles) → 1452 bp deleted

GGCAGGCAGGAAGGGAGGAGGAC------------------------ACCATGGCCCTCATGGACCTGGGTGAGGG

PARP3$^{-/-18}$ (both alleles) → 1454 bp deleted

GGCAGGCAGGAAGGGAGGAGGACCCCTTCCG-----------------------------TCATGGACCTGGGTGAGGGGTGAG

T98G

PARP3$^{-/-1}$ (both alleles) → 1515 bp deleted

GGCAGGCAGGAAGGGAGGAGGACCCCTTCCGCT-----------------------------GTGGGGCTGAGTCTCCCCACTCCCCT

PARP3$^{-/-2}$ (both alleles) → 1472 bp deleted

GGCAGGCAGGAAGGGAGGAGGACCCCTTCCGCTC------------------------GAGAGGCAGGCAGGGTGGCAGGGGCCTCA

U373-MG

PARP3$^{-/-a}$ (both alleles) → 1441 bp deleted

GGCAGGCAGGAAGGGAGGAGGACCCCTTCCGCTCCACCGC------------------GCCCTCATGGACCTGGGTGAGGG

PARP3$^{-/-b}$ (both alleles) → 1390 bp deleted

GGCAGGCAGGAAGGGAGGAGGACCCCTTCCGCTCCACCGCTGAGGC----------------CTCATCACTAACATCTTCAGCAAGGAG

Sup. Fig. 1: Sequencing analysis of the *PARP3*-deleted clones. Two PARP3$^{-/-}$ cell lines were established from LN229, T98G and U373-MG cell lines. The deleted sequences of the clones used are presented.

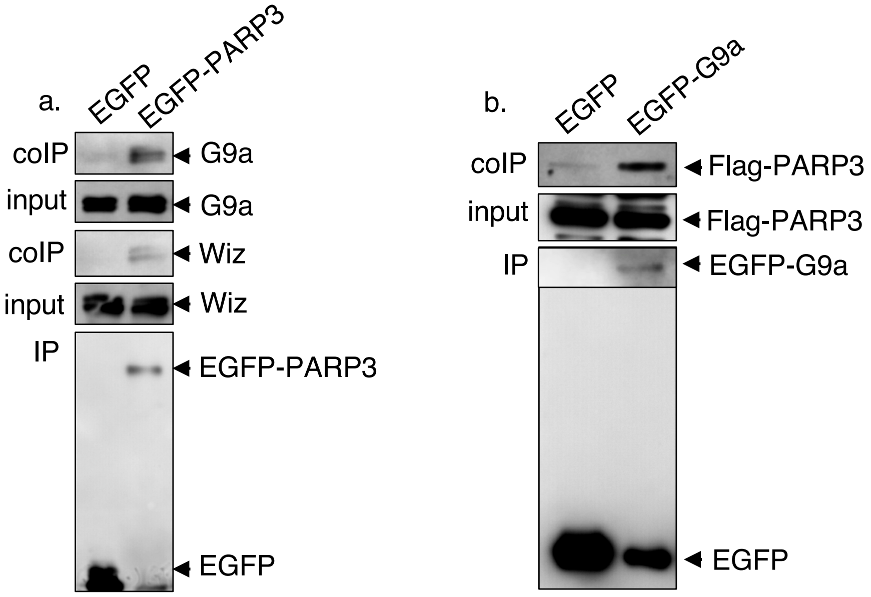

Sup. Fig. 2: PARP3 interacts with G9a. a. G9a and Wiz co-immunoprecipitate with EGFP-PARP3 but not EGFP. EGFP and EGFP-PARP3-expressing HEK293T cell extracts were immunoprecipitated with an anti-EGFP antibody and analysed by western blotting using anti-G9a, anti-Wiz and anti-EGFP antibodies. Input correspond to 1/40 of the total amount of cell extracts used for immunoprecipitation. **b**. Reverse IP: Flag-PARP3 co-immunoprecipitates with EGFP-G9a but not EGFP. $PARP3^{-/-3}$ LN229 cells with a stable expression of Flag-$PARP3^{WT}$ were transfected with either EGFP or EGFP-G9a. Cell extracts were immunoprecipitated with an anti-EGFP antibody and analysed by western blotting using anti-Flag and anti-EGFP antibodies. Input correspond to 1/43 of the total amount of cell extracts used for immunoprecipitation. Uncropped Western-blots are shown below.

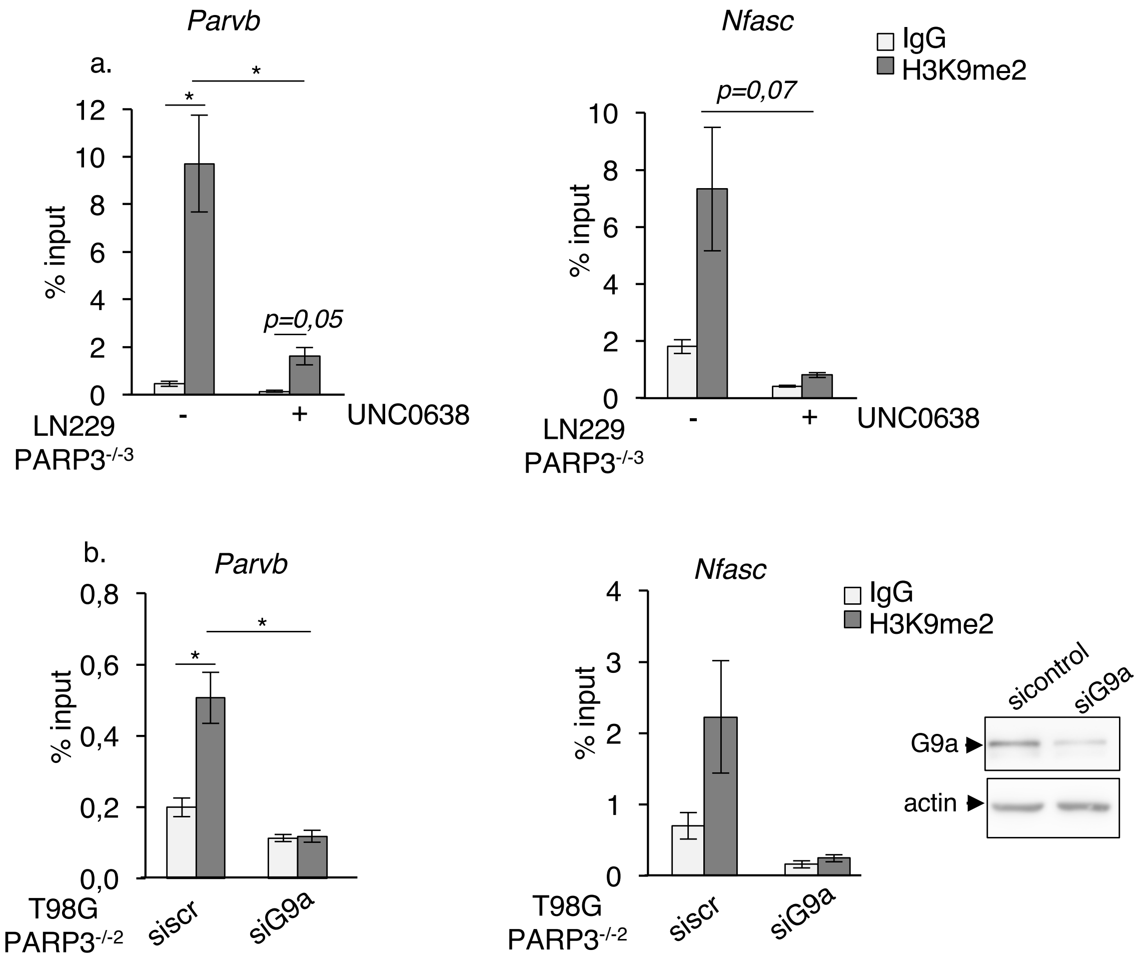

Sup. Fig. 3 The chemical inhibition of G9a or its silencing reduces the enrichment of H3K9me2 on the promoter of *Nfasc and Parvb* in PARP3-deficient cells. a. H3K9me2 binding was assessed on the promoter region of *Parvb* (left) *and Nfasc* (right) by ChIP-qPCR in the PARP3$^{-/-3}$ LN229 cells either mock-treated or treated with the selective G9a inhibitor UNC0638 (1 μM, 72h). IgG were used as ChIP negative controls. Data are represented as percent of input and are means (+/- s.e.m) of three replicates of a representative experiment. *p<0,05. **b**. H3K9me2 binding was assessed on the promoter region of *Parvb* (left) and *Nfasc* (right) by ChIP-qPCR in the PARP3$^{-/-2}$ T98G cells transfected with either sicontrol (siscr) or siG9a for 72h. IgG were used as ChIP negative controls. Data are represented as percent of input and are means (+/- s.e.m) of three replicates of a representative experiment. *p<0,05. Insert shows the efficiency of G9a depletion in the T98G PARP3$^{-/-2}$ cells.

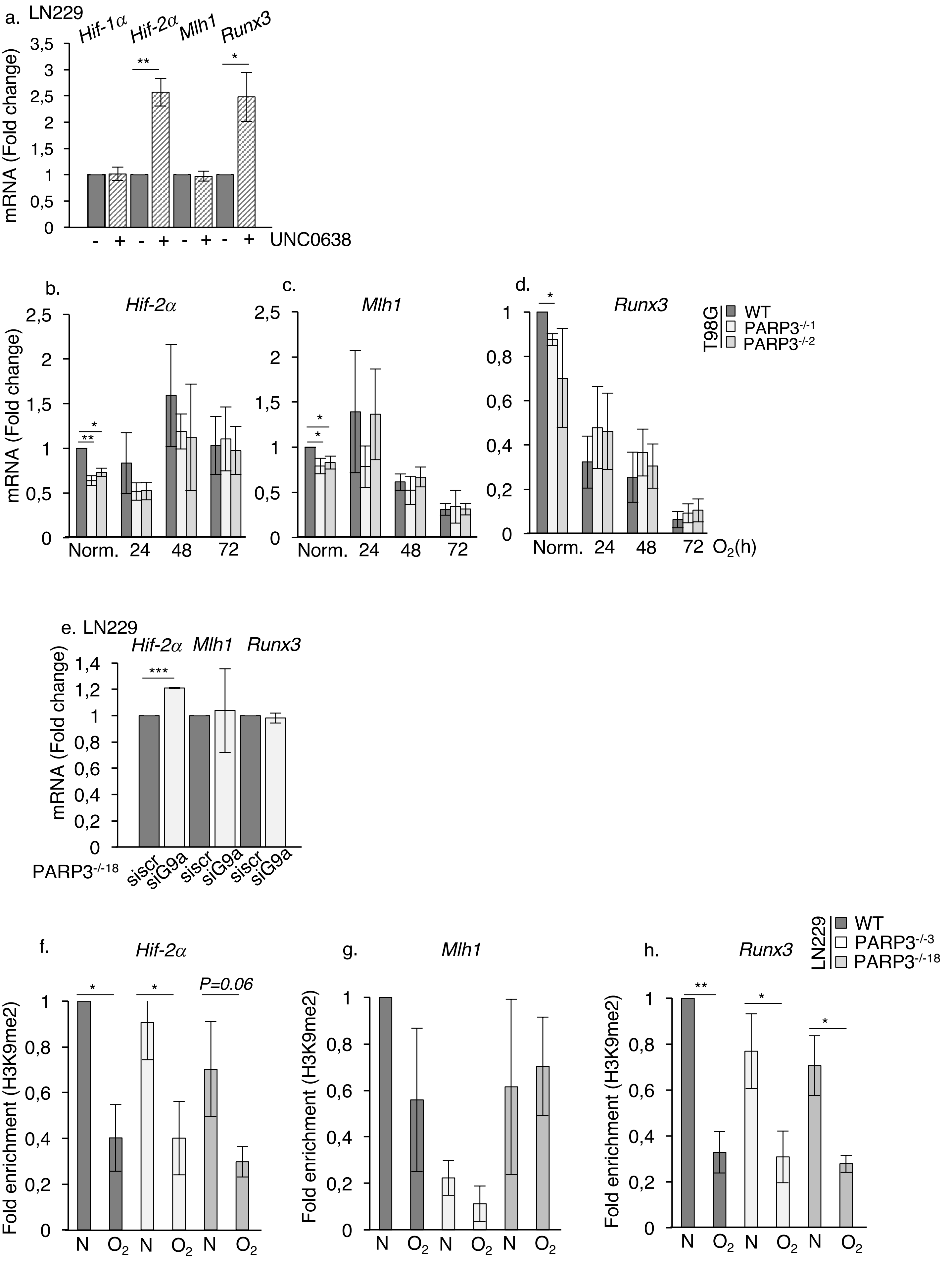

a. LN229
Hif-1α
Hif-2α
Mlh1
Runx3
mRNA (Fold change)
UNC0638
b.
Hif-2α
c.
Mlh1
d.
Runx3
Norm. 24 48 72 O2(h)
T98G
WT
PARP3-/-1
PARP3-/-2
e. LN229
Hif-2α Mlh1 Runx3
PARP3-/-18
siscr siG9a siscr siG9a siscr siG9a
f.
Hif-2α
P=0.06
g.
Mlh1
h.
Runx3
LN229
WT
PARP3-/-3
PARP3-/-18
Fold enrichment (H3K9me2)
N O2 N O2 N O2

Sup. Fig. 4: PARP3 regulates the hypoxia-responsive genes *Hif2α*, *Mlh1* and *Runx3* in common with G9a. a. The UNC0638-mediated chemical inhibition of G9a induces an upregulation of *Hif2α* and *Runx3* in LN229 cells. LN229 cells were either mock-treated or treated with the selective G9a catalytic inhibitor UNC0638 for 72h and then processed for RT-qPCR for *Hif-1α, Hif-2α, Mlh1* and *Runx3*. Data are expressed relative to *Actin*. Values represent means +/-s.e.m of three independent experiments. *p<0,05; **p<0,01. **b-d.** PARP3 deficiency in T98G cells induce the selective downregulation of *Hif-2a, Mlh1 and Runx3* in normoxia but not under 1% O_2-induced hypoxia. RT-qPCR expression analysis of *Hif-1a*, *Hif-2a*, *Mlh1* and *Runx3* in the parental WT, and two PARP3-deficient clones of T98G cells grown under normal conditions or under 1% O_2 for the indicated time points. Data are expressed relative to *Actin*. Values represent means +/- s.e.m. of 3 independent experiments and 3 technical replicates. **e**. Recovery of expression of *Hif-2α* in the $PARP3^{-/-18}$ cells upon G9a silencing. The $PARP3^{-/-18}$ LN229 cells were transfected with either sicontrol or siG9a for 72h. The transcript levels of *Hif-2α, Mlh1* and *Runx3* were analysed by RT-qPCR. Data are expressed relative to *Actin*. Values represent means +/- s.e.m. of 3 independent experiments and 3 technical replicates. ***p<0,001. **f-h.** The absence of PARP3 does not impair the enrichment of H3K9me2 onto *Hif-2α, Mlh1* and *Runx3*. H3K9me2 binding was assessed on the promoter region of *Hif-2α, Mlh1* and *Runx3* by ChIP-qPCR in the parental WT versus the $PARP3^{-/-3}$ and $PARP3^{-/-18}$ LN229 cells grown in normoxia (N) or exposed to 1% O_2 for 72h. Enrichments were normalized to an IgG antibody used as ChIP negative control and fold inductions were calculated relative to the parental WT LN229 grown in normoxia. Data are represented a percent of input and are means (+/- s.e.m) of three biological replicates. *p<0,05, **p<0,01.

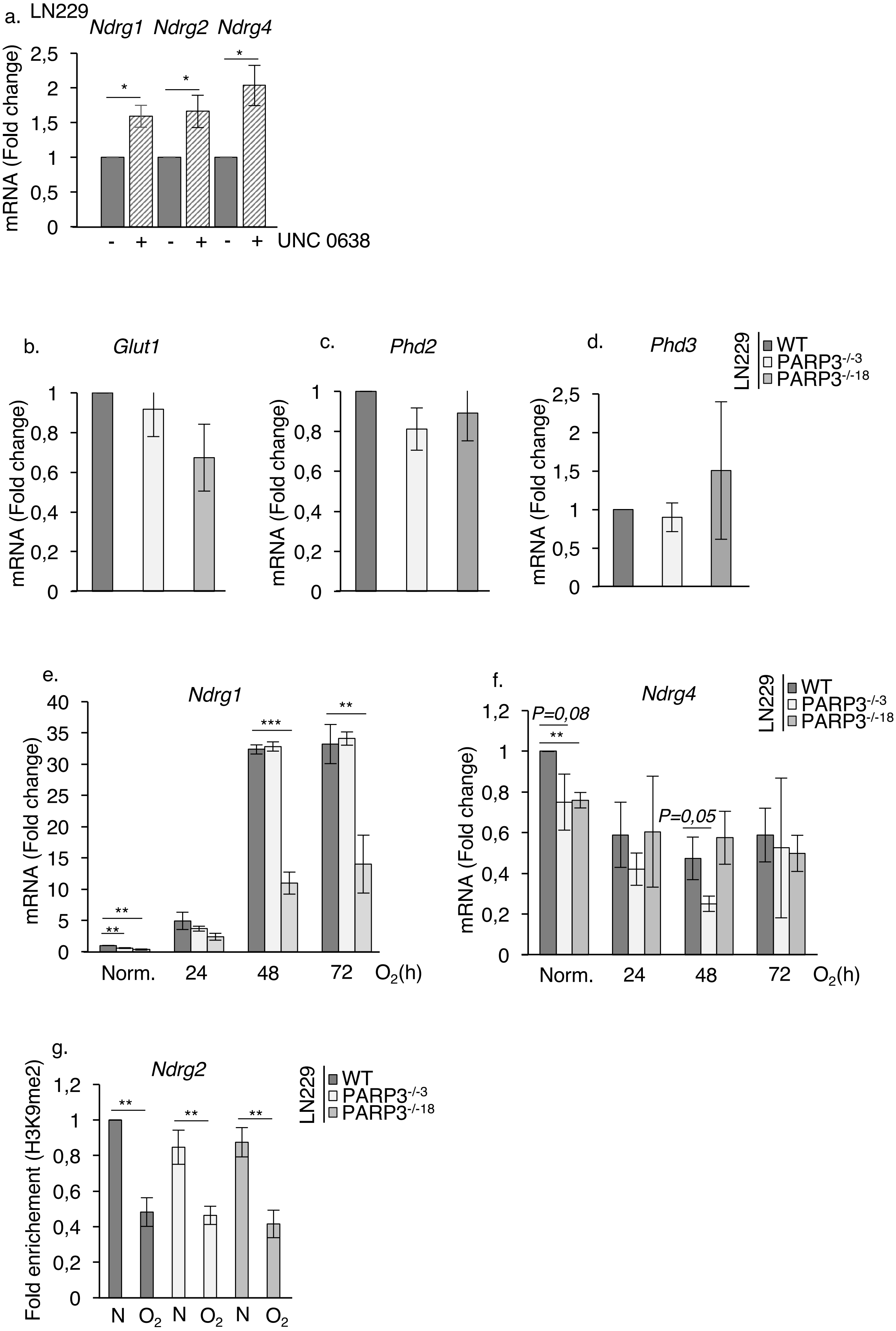
a. LN229
Ndrg1 Ndrg2 Ndrg4
mRNA (Fold change)
UNC 0638
b. Glut1
c. Phd2
d. Phd3
LN229
WT
PARP3-/-3
PARP3-/-18
e. Ndrg1
f. Ndrg4
P=0,08
P=0,05
Norm. 24 48 72 O2(h)
g. Ndrg2
Fold enrichement (H3K9me2)
N O2 N O2 N O2

Sup. Fig. 5: PARP3 regulates the hypoxia-responsive genes *Ndrg1*, *Ndrg2*, *Ndrg4* in common with G9a. a. The chemical inhibition of G9a induces an upregulation of *Ndrg1, Ndrg2* and *Ndrg4* in LN229 cells. LN229 cells were either mock-treated or treated with the selective G9a catalytic inhibitor UNC 0638 for 72h and then processed for RT-qPCR for *Ndrg1, Ndrg2* and *Ndrg4*. Data are expressed relative to *Actin*. Values represent means +/-s.d of three independent experiments. **b-d.** The absence of PARP3 does not alter the expression of *Glut1*, *Phd2*, *Phd3*. RT-qPCR for *Glut1*, *Phd2*, *Phd3* in WT, $PARP3^{-/-3}$ and $PARP3^{-/-18}$ LN229 cells cultured under normoxia. Data are expressed relative to *Gapdh*. Values represent means +/-s.d of three independent experiments. **e-f.** Impact of the absence of PARP3 on the expression of *Ndrg1* and *Ndrg4* under hypoxia. RT-qPCR expression analysis of *Ndrg1* and *Ndrg4* in the parental WT, $PARP3^{-/-3}$ and $PARP3^{-/-18}$ LN229 cells grown under normal conditions or under 1% O_2 for the indicated time points. Data are expressed relative to *Actin*. Values represent means +/- s.e.m. of 3 independent experiments and 3 technical replicates. *p<0,05, **p<0,01, ***p<0,001. **g**. The absence of PARP3 does not impair the enrichment of H3K9me2 onto *Ndrg2*. H3K9me2 binding was assessed on the promoter region of *Ndrg2* by ChIP-qPCR in the parental WT versus the $PARP3^{-/-3}$ and $PARP3^{-/-18}$ LN229 cells grown in normoxia (N) or exposed to 1% O_2 for 72h. Enrichments were normalized to an IgG antibody used as ChIP negative control and fold enrichments were calculated relative to the parental WT LN229 grown in normoxia. Data are represented a percent of input and are means (+/- s.e.m) of three biological replicates. *p<0,05, **p<0,01.

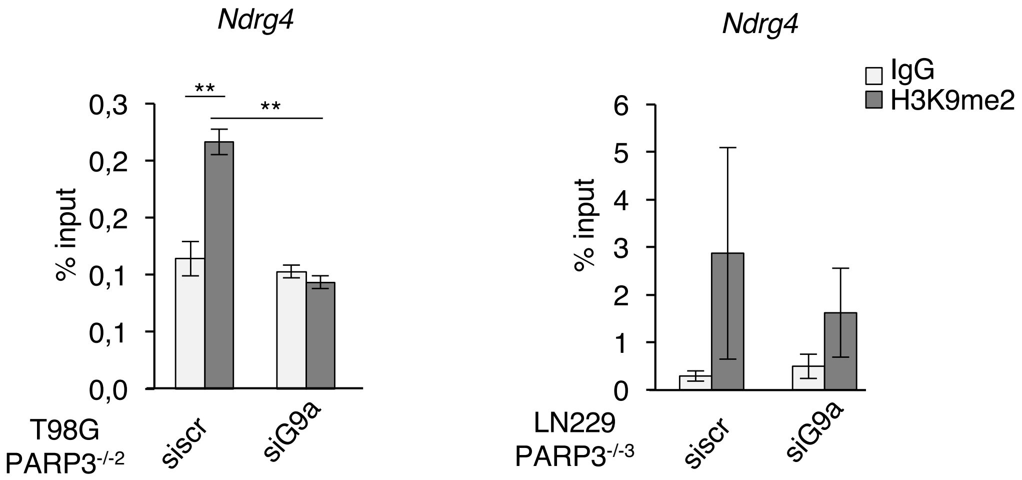

Sup. Fig. 6. The depletion of G9a reduces the enrichment of H3K9me2 on the promoter of *Ndrg4* in PARP3-deficient T98G cells. H3K9me2 binding was assessed on the promoter region of *Ndrg4* by ChIP-qPCR in the PARP3$^{-/-2}$ T98G (*left*) and PARP3$^{-/-3}$ LN229 (*right*) cells transfected with either sicontrol (siscr) or siG9a for 72h. IgG were used as ChIP negative controls. Data are represented as percent of input and are means (+/- s.e.m) of three replicates of a representative experiment. **p<0,01.

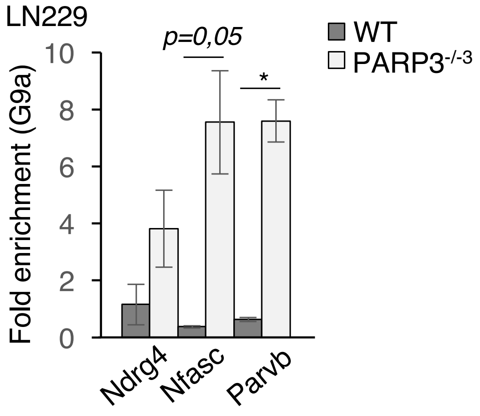

Sup. Fig. 7. Increased enrichment of G9a on the promoter of *Ndrg4, Nfasc and Parvb* in PARP3-deficient LN229 cells. G9a binding was assessed on the promoter region of *Ndrg4, Nfasc and Parvb* by ChIP-qPCR in the parental WT versus the PARP3$^{-/-3}$ LN229 cells. Enrichments were normalized to an IgG antibody used as ChIP negative control and fold enrichments were calculated relative to the parental LN229. Data are are means (+/- s.e.m) of three replicates of a representative experiment. *p<0,05.

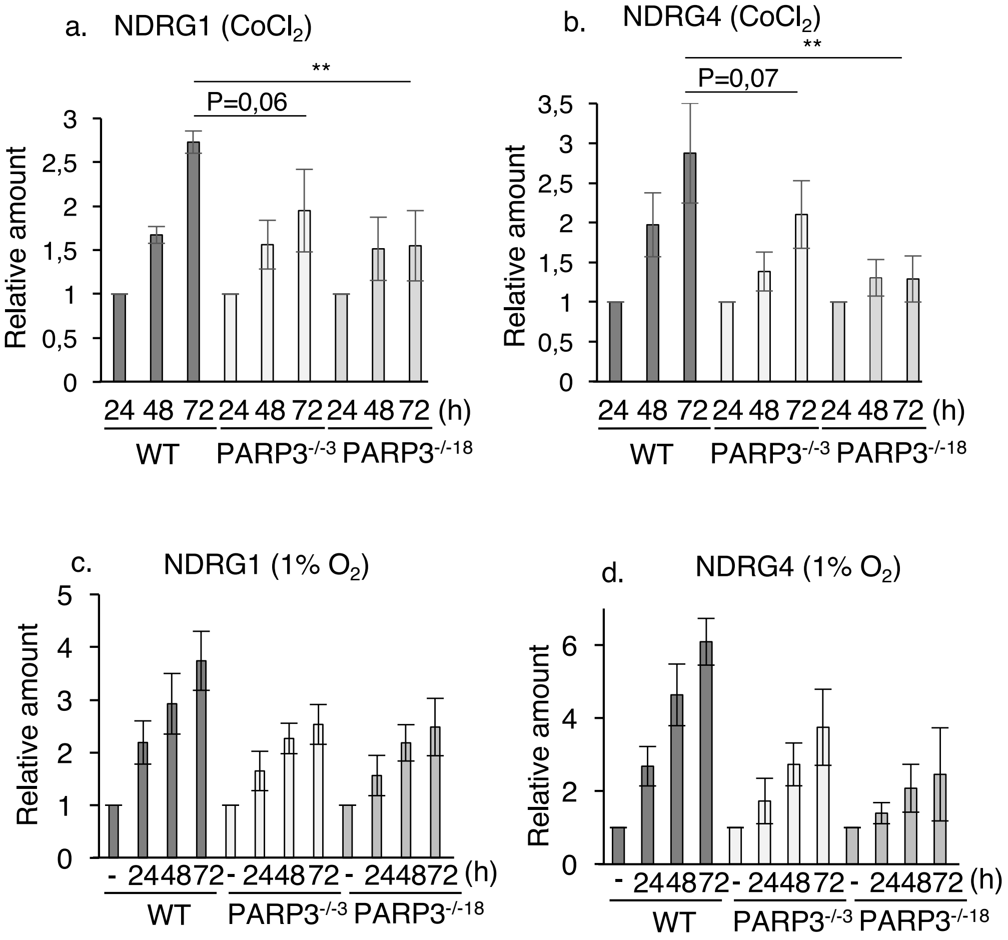

Sup. Fig. 8: The absence of PARP3 impairs efficient induction of NDRG1 and NDRG4 under hypoxia a-d. Bar graphs depicts the relative signal intensities of NDRG1 versus actin and NDRG4 versus actin throughout time upon exposure to $CoCl_2$ or 1% O_2, measured in up to 5 independent experiments using Image J. Mean values +/-s.d are indicated. *p<0,05; **p<0,01.

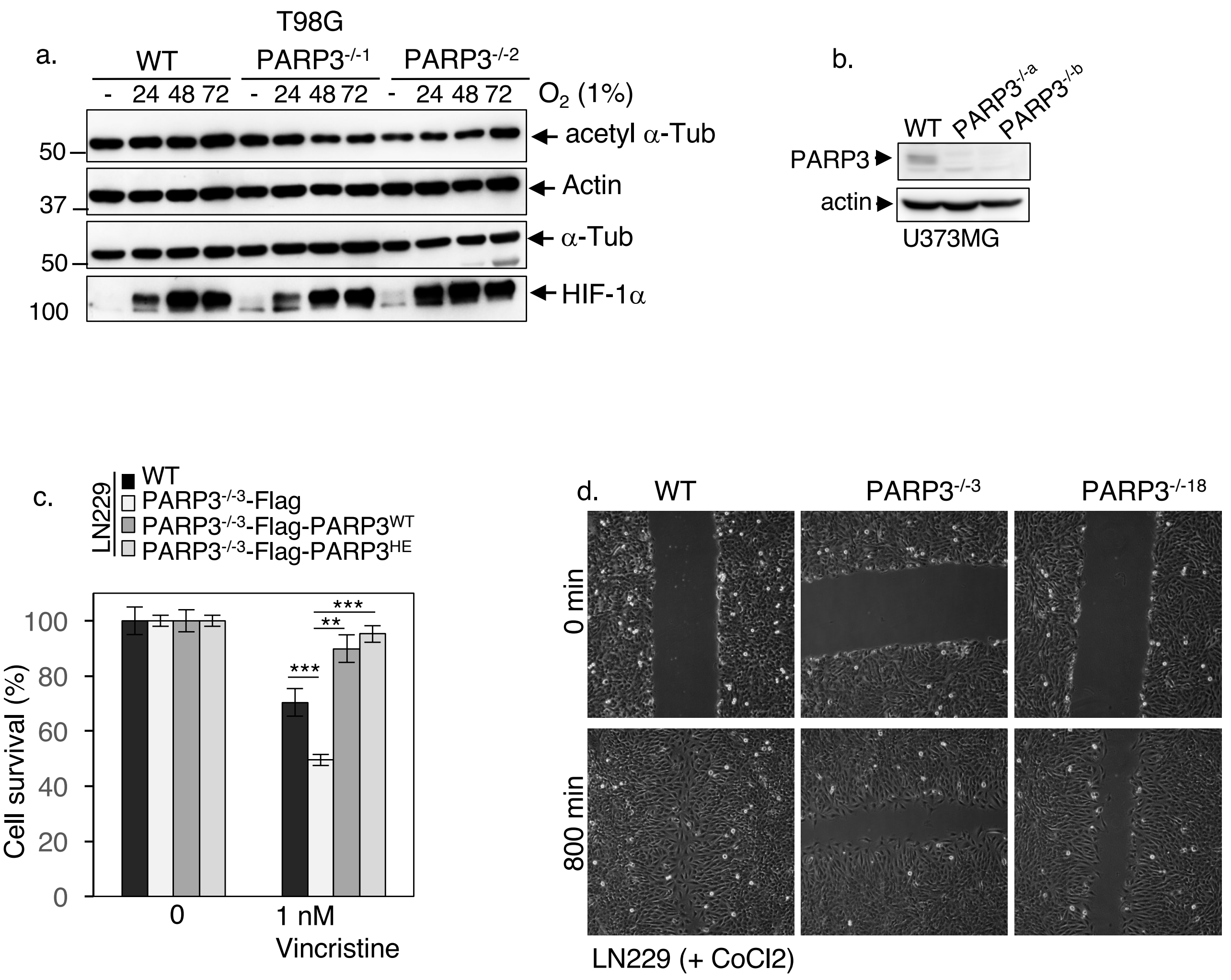

Sup. Fig. 9: The absence of PARP3 compromizes cell response to hypoxia. a. PARP3 deficiency reduces tubulin acetylation under 1% O_2-induced hypoxia in T98G cells. The parental WT, PARP3$^{-/-1}$ and PARP3$^{-/-2}$ T98G cells were cultured under 1%O_2-induced hypoxia for the indicated time points. Acetyl α-Tubulin, actin, α-Tubulin, HIF-1α were analysed by western blotting using the appropriate antibodies. **b**. Western blot analysis of PARP3 expression in the wild-type (WT) and two PARP3$^{-/-a}$, PARP3$^{-/-b}$ U373MG clones selected upon screening and sequence analysis. Actin is used as control. Uncropped Western-blots are shown below. **c**. Sensitivity to vincristine in the PARP3 knockout LN229 cells is rescued by the re-expression of PARP3. Survival is expressed as the percentage of colonies formed in the PARP3$^{-/-3}$ LN229 cells with a stable expression of either the Flag control (Flag), Flag-PARP3^{WT} or Flag-PARP3^{HE} versus the parental LN229 (WT) set to 100%. Mean values of technical triplicates +/- s.d. of a representative experiment are indicated. **$p<0,01$; ***$p<0,001$. **d.** PARP3 deficiency alters cell motility under hypoxic stress. Light microscopy of representative areas of scratch assays on cultures of WT, PARP3$^{-/-3}$ and PARP3$^{-/-18}$ LN229 cells maintained under chemical-induced hypoxia (100 μM CoCl2, 48h). Times (min) post-scratch are shown.

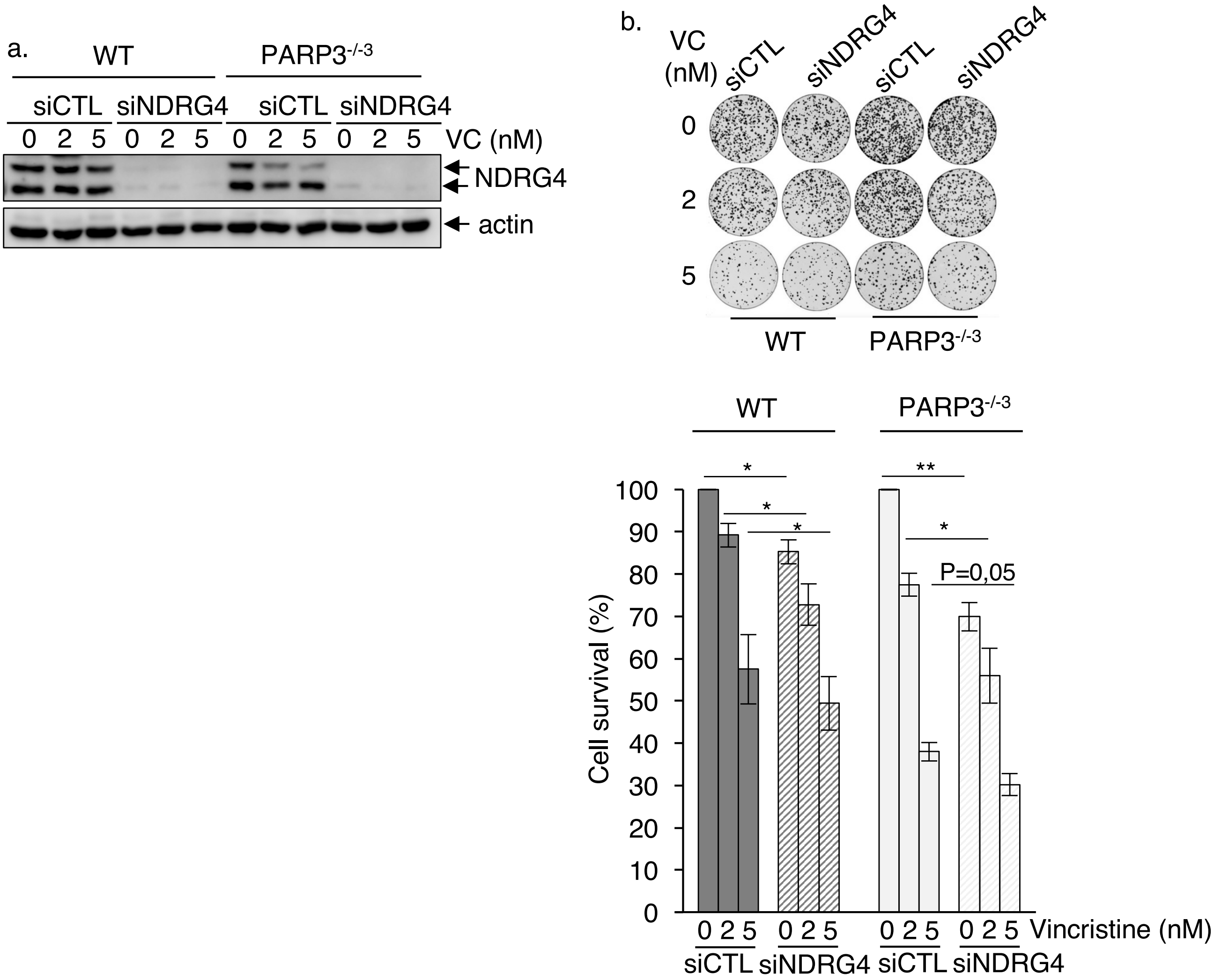

Sup. Fig. 10. The depletion of NDRG4 weakly enhances the sensitivity of PARP3$^{-/-3}$ LN229 to vincristine. a. Western blot analysis for the expression of NDRG4 and actin in sicontrol (siCTL) and siNDRG4-treated WT and PARP3$^{-/-3}$ cells exposed to the indicated doses of vincristine. **b**. Dose response clonogenic survival curves of the sicontrol or siNDRG4-treated wild type (WT) and PARP3$^{-/-3}$ LN229 cell lines exposed to the indicated doses of vincristine. Results represent the mean values of three independent experiments +/- s.d. *p<0,05, **p<0,01.

Material and methods

Reagents

Gene specific siRNA for NDRG4 (ON_TARGET plus human NDRG4 L-006937-00) and negative control siRNA were obtained from Horizon Discovery. Cells were transfected with 25 nM siRNA using JetPrime (PolyPlus Transfection) according to the manufacturer's instructions and processed 72h later.

The G9a selective inhibitor UNC0638 was purchased from Sigma-Aldrich. Cells were treated for 72h at 1 μM.

Suplementary Table 1: List of antibodies used in the study

Name	Company	Concentration
Rabbit anti-Flag (F7425)	Sigma-Aldrich	WB 1:2000
Rabbit anti-G9a (ab185050)	Abcam	WB 1:1000
Rabbit anti-GLP1 (09-078)	Millipore	WB 1:1000
Rabbit anti-Wiz (NBP2-58557)	Novusbio	WB 1:500
Mouse anti-EGFP (11814460001)	Roche	WB 1:1000
Mouse anti-acetyl α-tubulin (T6793)	Sigma-Aldrich	WB 1:1000, IF 1:200
Rabbit anti-detyr α-tubulin	Millipore	WB 1:500
Mouse anti-α tubulin (T9026)	Sigma-Aldrich	WB 1:500
Rabbit anti-HIF-1α (A300-286A)	Bethyl Laboratories	WB 1:2000
Rabbit anti-HIF-2α (NB100-122)	Novusbio	WB 1:1000
Rabbit anti-NDRG1 (42-6200)	Thermos Fisher Scientific	WB 1:1000
Rabbit anti-NDRG4 (HPA015313)	Sigma-Aldrich	WB 1:500
Mouse anti-β tubulin (sc-166729	Santa Cruz Biotech	WB 1:1000
Alexa Fluor^{TM} 790 Goat anti-Mouse	Life Technologies	IF 1:10000
Goat anti-rabbit IgG HRP conjugated (A120-101P)	Bethyl Laboratories	WB 1:30000
Goat anti-mouse IgG HRP conjugated (A90-116P)	Bethyl Laboratories	WB 1:30000
Alexa Fluor-568 Phalloidin (A11037)	Invitrogen	IF 1:50
Mouse anti-H3K9me2 CHIP grade (ab1220)	Abcam	ChIP: 4 μg / 50 μg chromatin
Rabbit anti-G9a/EHMT2 (3306S)	Cell Signaling technology	ChIP: 7 μl / 10 μg chromatin
Control IgG (A900-117A)	Bethyl Laboratories	ChIP: 4 μg / 50 μg chromatin

Suplementary Table 2: List of primers used in the study

Primers for qPCR

Gene	Forward	Reverse s
ACTIN	TCACCCACACTGTGCCCATCTACG A	CAGCGGAACCGCTCATTGCCAATG G
ADAMTS 4	TCATCACTGACTTCCTGGACAA	GAAAGTCACAGGCAGATGCA
COL22A1	GTGATTGGCAAGCGCCTCTAC	CAAGTCTCCAATTCTGCGTGTCTC
CPXM2	GTGCGCGGGAAGAAATGAC	CCTCCCTTGAGTGATGACACC
FAIM2	GACTCATCCTGGCCATCCTCCTAC	GGCGTCGGTTACCCATCAGC
FRMD4B	TCGAAGTAGAGAGCGAAACCATC	CTTGACCTCGAGTGAGACCTTTG
GAPDH	AGCCACATCGCTCAGACA	GCCCAATACGACCAAATCC
GLUT1	GGCCTTTTCGTTAACCGCTT	AGCATCTCAAAGGACTTGCCC
HIF-1α	CATAAAGTCTGCAACATGGAAGG	ATTTGATGGGTGAGGAATGGGTT
HIF-2α	GCGCTAGACTCCGAGAACAT	TGGCCACTTACTACCTGACCCTT
KCNA2	AGAGCGTCCTCTGCCTGAAA	TCCCGGAAGATGGGCAATGT
KCNK5	ACCACCCACTCATCTTCCAG	AGTGCTGGTGAAGGTGGACT
KCNK9	GCAAGGCCTTCTGCATGTTC	TTCAGCAGGTAGCGCACGAA
NDRG1	GGCAACCTGCACCTGTTCATCAAT	TGAGGAGAGTGGTCTTTGTTGGGT
NDRG2	CCCTGTGTTCCCTTTGGGAT	GTGAGGCCTGTTAGCTTGTG
NDRG4	GGAGGTTGTCTCTTTGGTCAAGGT	CTCATGACAGCAGCCACCAGAAT
NFASC	AACGCCTTTGTCAGTGTGCT	TAGTTGCCACCATCCAGGTT
PARVB	CATCCGCCTTCCTGAGCAT	AGCAGGCCTTCCCGTTTC
PHD2	GCACGACACCGGGAAGTT	CCAGCTTCCCGTTACAGT
VWA5A	TGCTTCTGCCCCATTGAAGA	CTGTGCTGGTCCTTGTGAC
MLH1	CAGGTATTCAGTACACAATGCAGG C	CTACCAGACGATGGTTGATGAAGA G
RUNX3	TCTGTAAGGCCCAAAGTGGGTA	ACCTCAGCATGACAATATGTCACA A
PHD3	GGCCATCAGCTTCCTCCTG	GGTGATGCAGCGACCATCA
G9a	TACACCACTCATTGGGGATG	GGGAAGAGGGGAATGACTTT

Primers for qPCR (Taqman)

Gene	Reference (Thermofisher)
ACAN	Hs00153936_m1
ACTIN	Hs01060665_m1
FBLN5	Hs00197064_m1
LYN	Hs00176719_m1
GAPDH	4326317E-0509010
WNT10B	Hs00559664_m1

Primers for ChIP

Gene	Forward (5’….3’)	Reverse (5’….3’)
NFASC	TTCTCTTCCCCATCTCATGC	TGAGAGGGACTGTGATGCAG
PARVB	CGTTCATTCCATGAGCAGTG	CGTGAGCACACGCAGTAAAA
NDRG4	GGTCCGTCCGGGACTAGC	GAGATGCGGACGAGACAGAC
NDRG2	GGCATTGACCCCAGAGTCCCTG	GAAGTTGGACAACAAGGCGGGG
RUNX3	GGTTGCAGAAGTCACAGG	AATTTGCTTAGAACGTCCG
MLH1	ACCGCTCGTAGTATTCGTGCTC	GTGGATGACGCCCAAAAGAAG
HIF-2α	ACTCCTGGTCACCCCTCAAG	TTTCTGGGAGCTCAGAATGG

Primers for PCR and sequencing

Gene	Forward (5'….3')	Reverse (5'….3')
PARP3	GCTGGGTGTGGTCTGGGGCTGGGAATG	CGAAGTTGTGCGGGATGACG

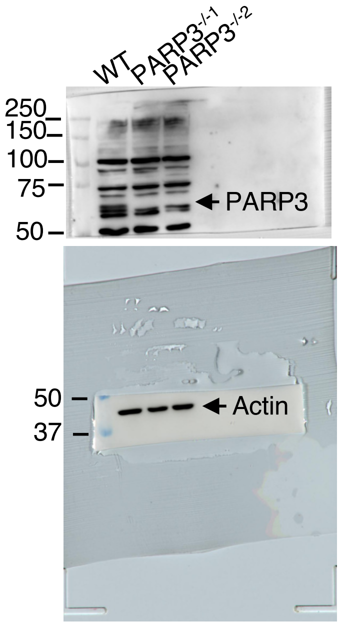

Figure 1b: Uncropped blots showing the expression of PARP3 and actin in WT, PARP3$^{-/-1}$ and PARP3$^{-/-2}$ T98G cells. For clearer results, the membrane was cut before hybridization with both antibodies.

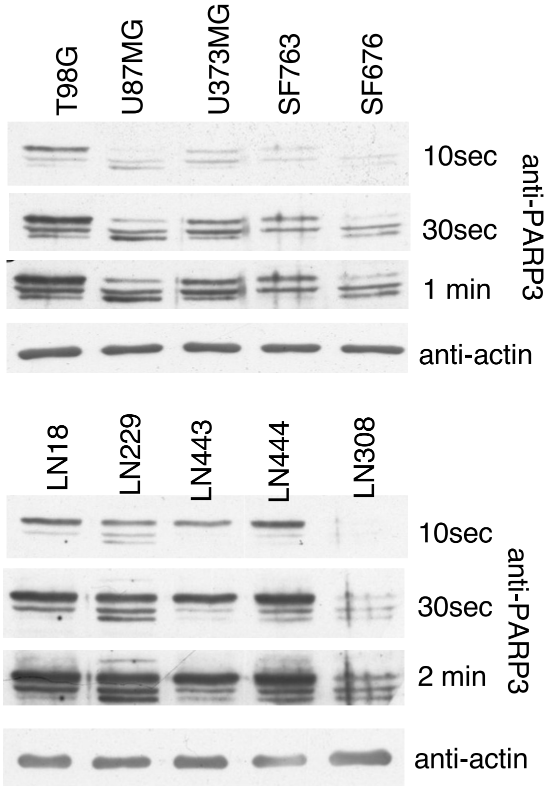

Figure 1a: Original revelations showing the expression of PARP3 and actin in different cell lines. For the bottom images, actin has been revealed on a separate membrane. The uncropped membranes are not available, the original files were lost.

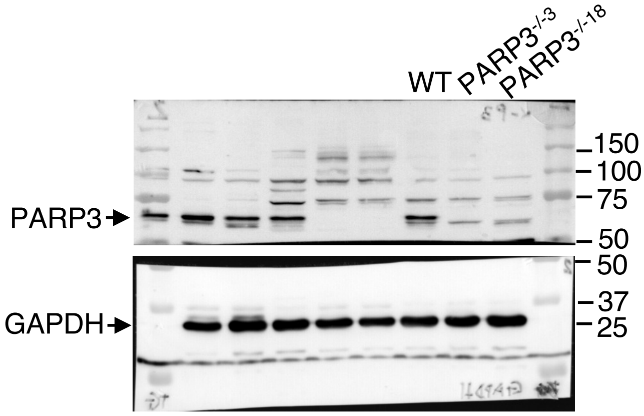

Figure 1d (upper panel): Uncropped blots showing the expression of PARP3 and GAPDH in WT, PARP3$^{-/-3}$ and PARP3$^{-/-18}$ LN229 cells. For clearer results, the membrane was cut before hybridization with both antibodies.

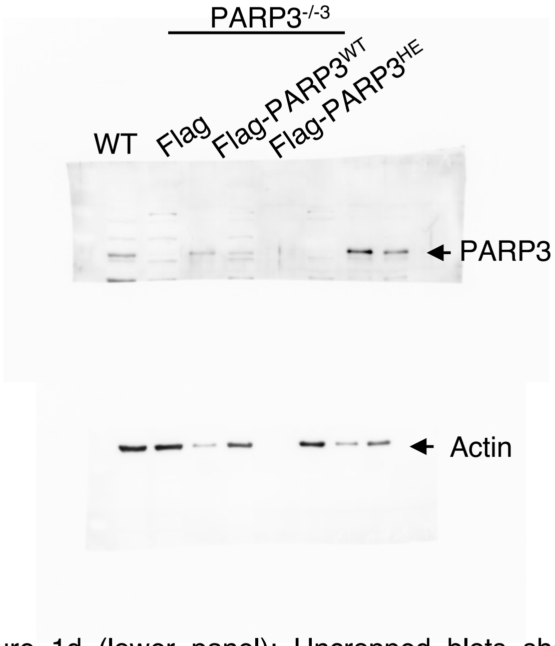

Figure 1d (lower panel): Uncropped blots showing the expression of PARP3 and Flag-PARP3 fusion proteins and actin in WT and PARP3$^{-/-3}$ complemented LN229 cells. For clearer results, the membrane was cut before hybridization with both antibodies.

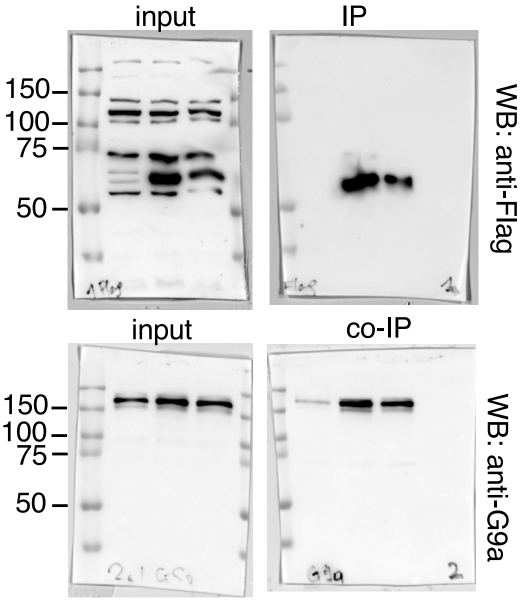

Figure 2a: Uncropped blots showing the expression of Flag-PARP3 and G9a in the inputs and after anti-Flag immunoprecipitation.

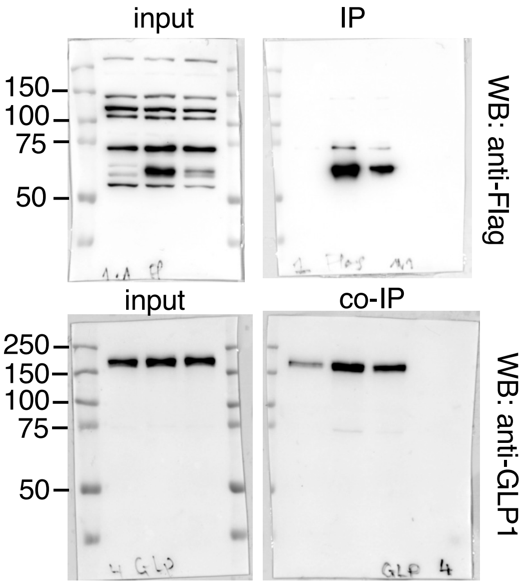

Figure 2b: Uncropped blots showing the expression of Flag-PARP3 and GLP1 in the inputs and after anti-Flag immunoprecipitation.

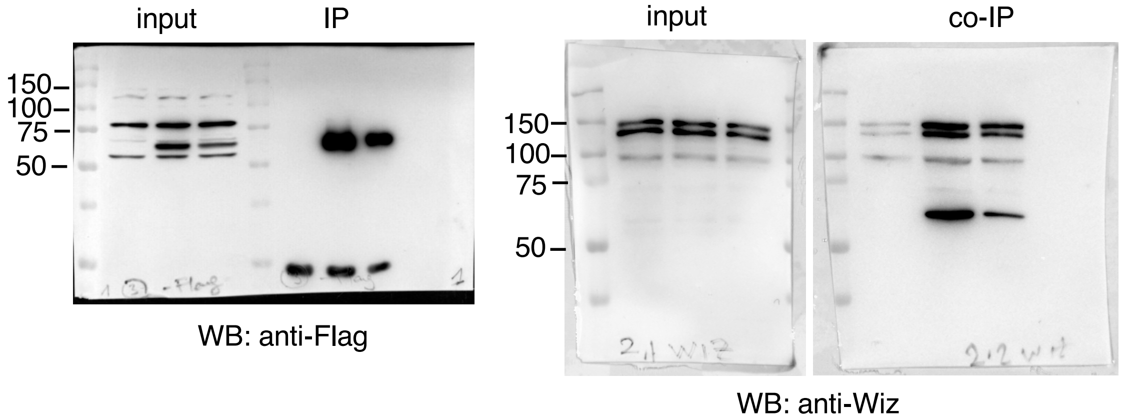

Figure 2c: Uncropped blots showing the expression of Flag-PARP3 and Wiz in the inputs and after anti-Flag immunoprecipitation.

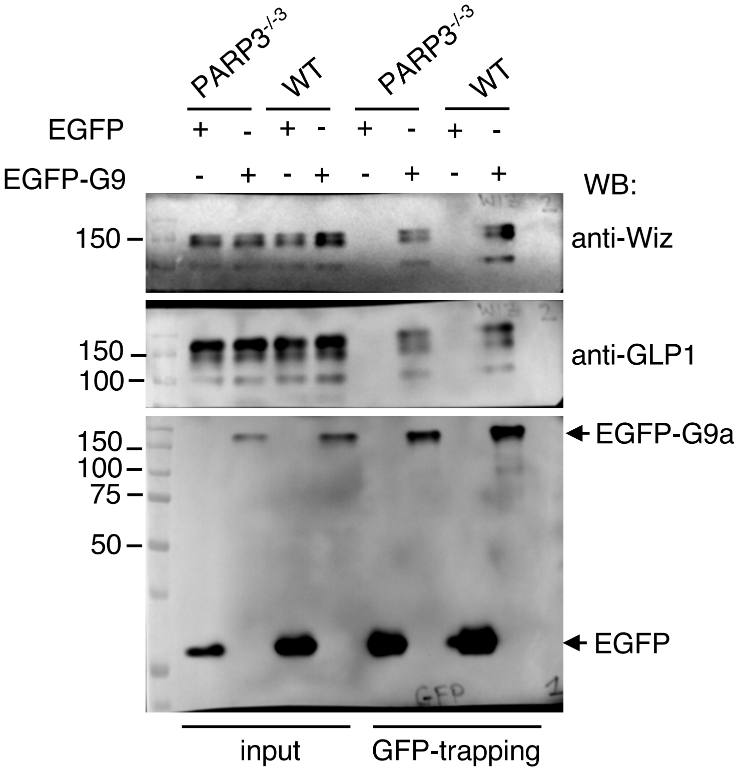

Figure 2d: Uncropped blots showing the expression of EGFP, EGFP-G9a, Wiz and GLP1 in the inputs and after GFP trapping. The same membrane was used for successive hybridization with anti-Wiz followed by anti-GLP1 antibodies. The gel was cut around 75 Kda for an other use.

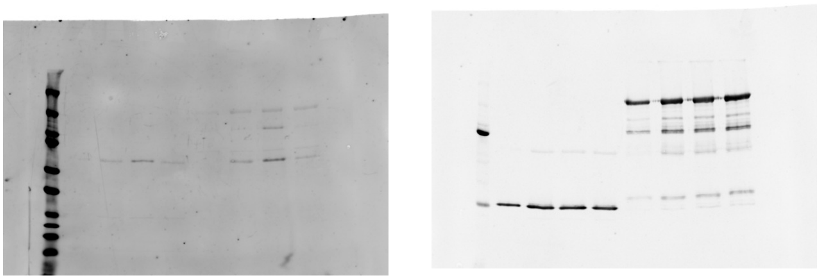

Figure 2e: Uncropped blots showing the ADPRylated proteins (left side). Proteins modified by biotin-NAD$^+$ were detected using streptavidin-Alexa 680. The expression of EGFP and EGFP-G9a fusion proteins (right side) were detected by anti-EGFP immunoblotting. Membrane edges are not visible because the window selected for analysis was cropped close to the samples to avoid large files.

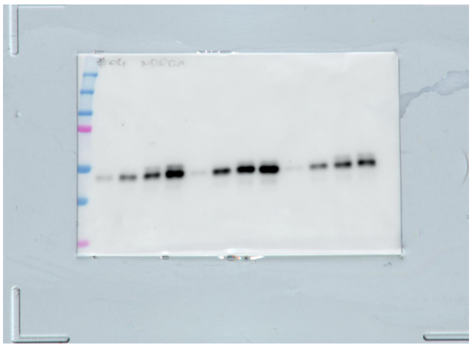

WB: NDRG1

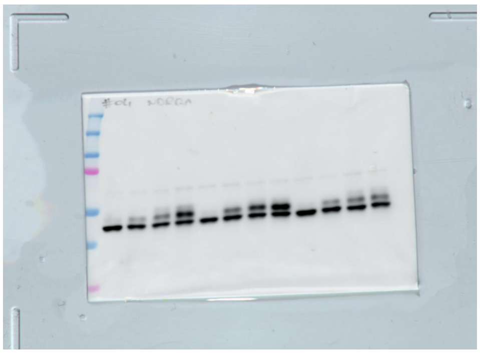

WB: actin

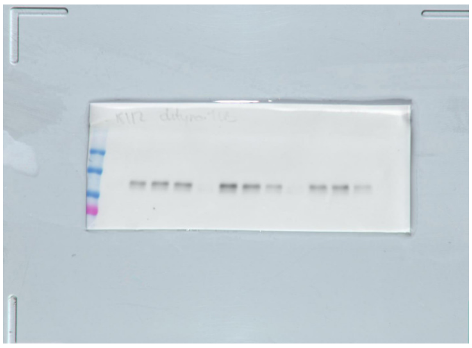

WB: HIF1α

Figure 6h (upper panel): Uncropped blots showing the expression of NDRG1, actin and HIF1α. NDRG1 and actin were revealed on the same membrane. HIF1α was revealed on a separate membrane that was cut for an other use. All proteins from Figure 6h, have been analysed using the same extract (biological replicate).

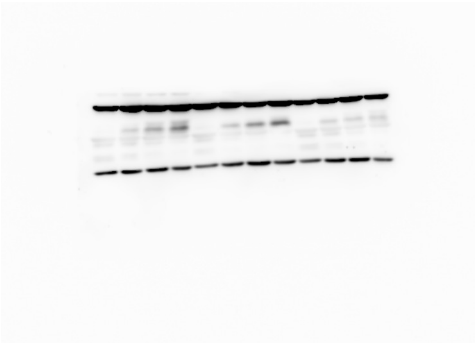

WB: NDRG4

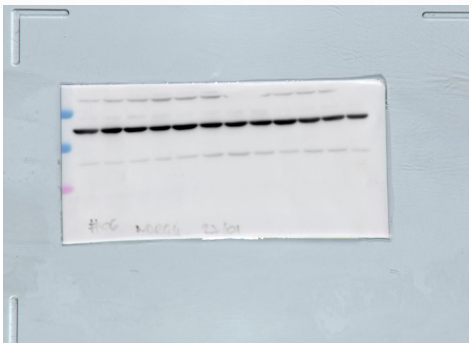

WB: actin

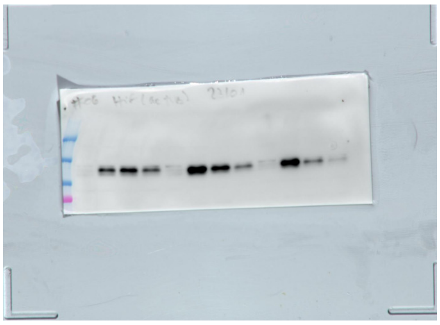

WB: HIF1α

Figure 6h (lower panel): Uncropped blots showing the expression of NDRG4, actin and HIF1α. NDRG4 and actin were revealed on the same membrane. HIF1α was revealed on a separate membrane that was cut for an other use. All proteins from Figure 6h, have been analysed using the same extract (biological replicate).

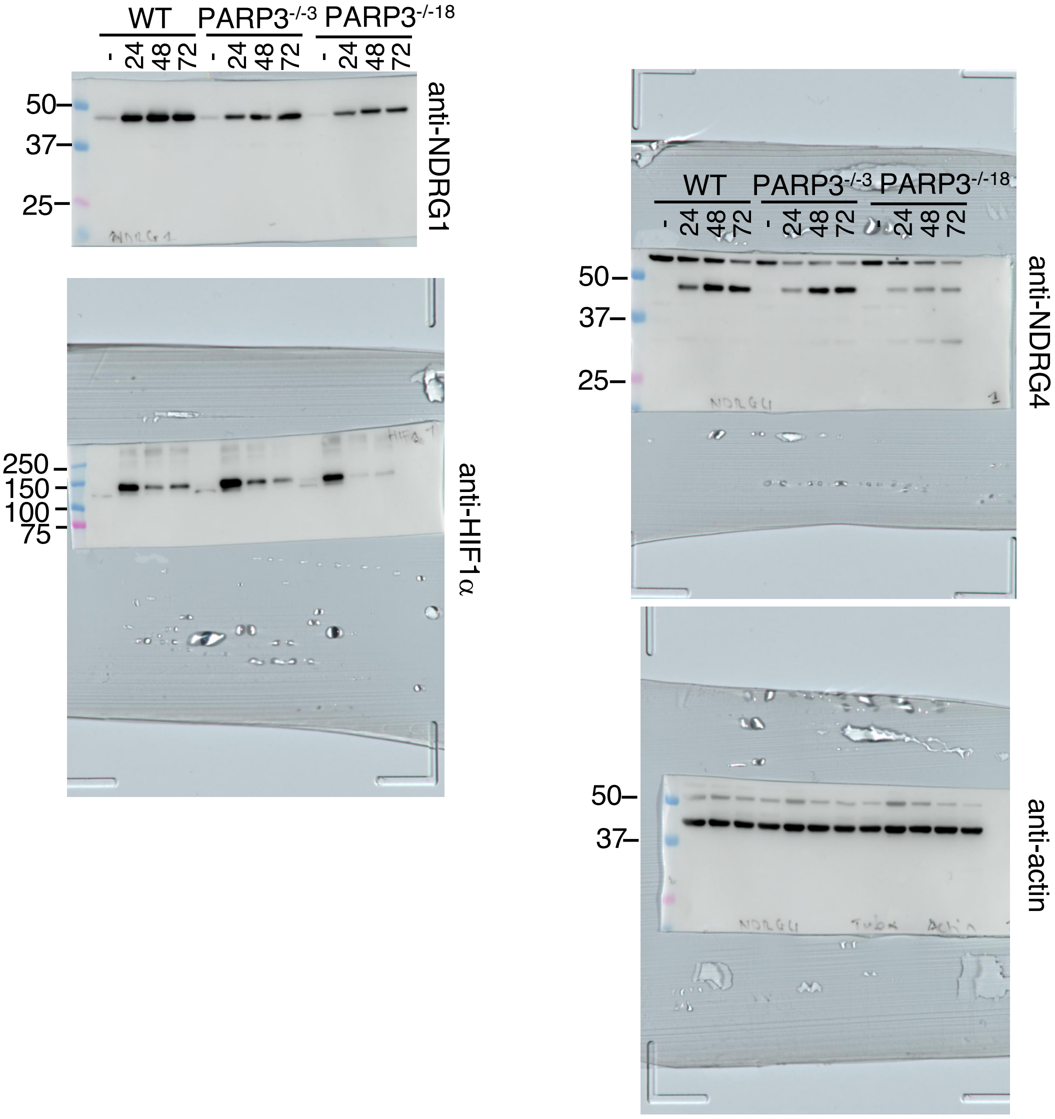

Figure 6i: Uncropped blots showing the expression of NDRG1, NDRG4, HIF1α and actin. All proteins from Figure 6i, have been analysed using the same extract (biological replicate). For clearer results, the membranes were cut before hybridization with antibodies.

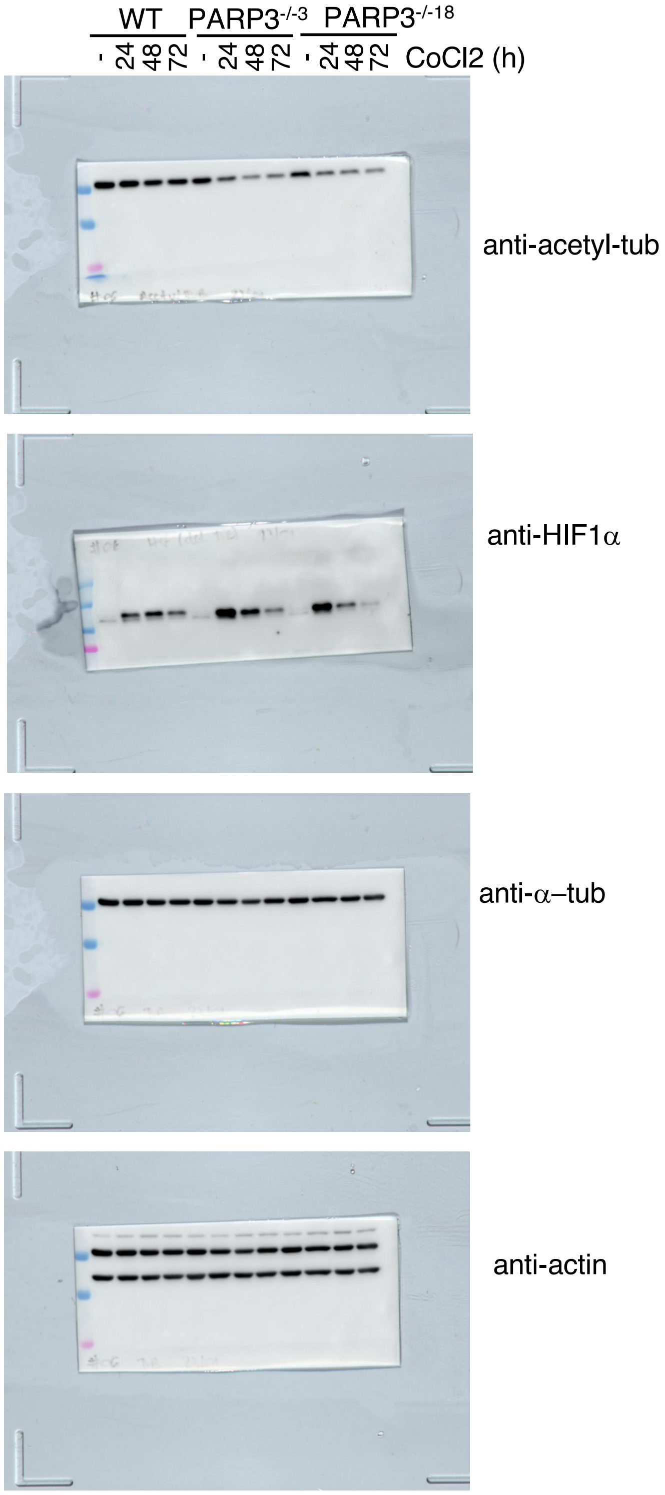

Figure 7a: Uncropped blots showing the expression of acetyl-tub, HIF1α, α-tub and actin. All proteins from Figure 7a have been analysed using the same extract (biological replicate). For clearer results, the membranes were cut before hybridization with antibodies.

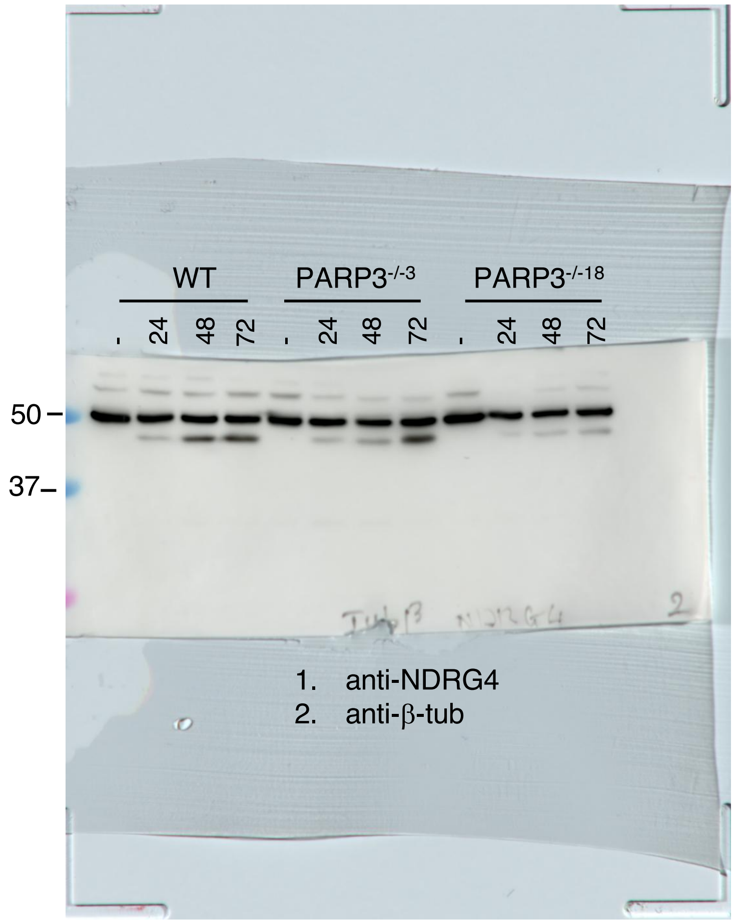

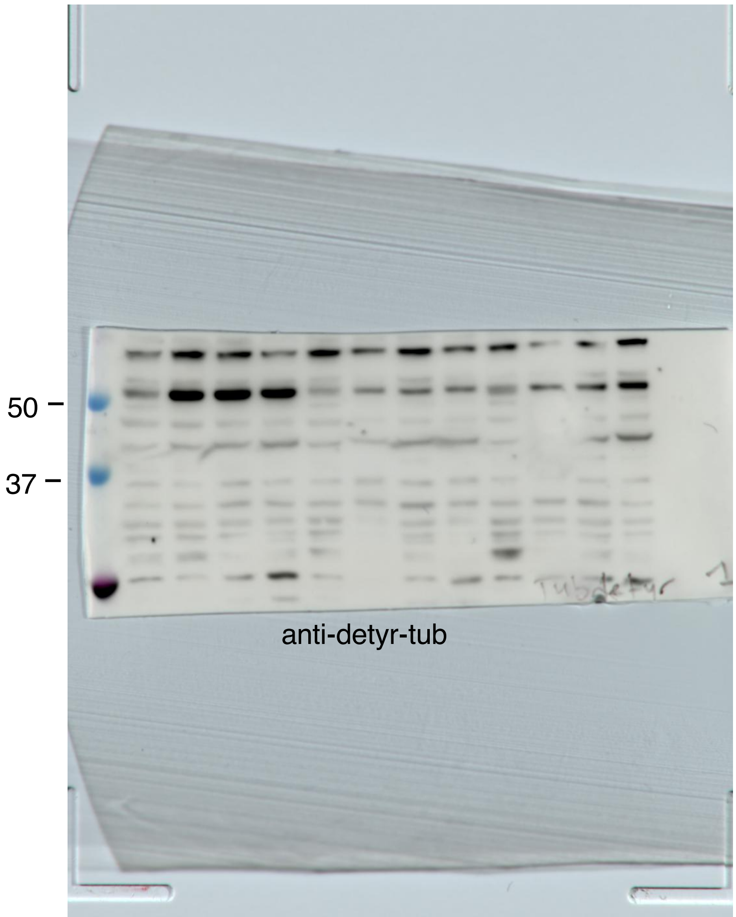

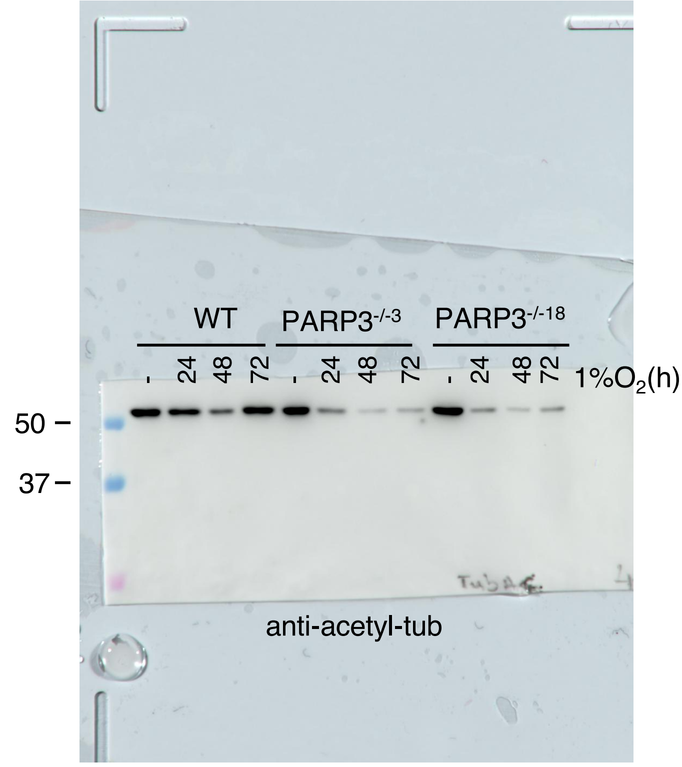

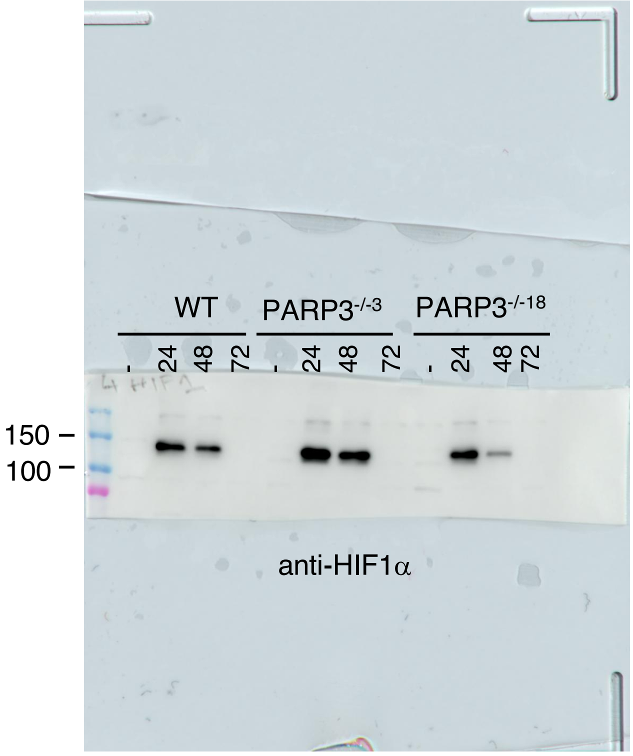

Figure 7c: Uncropped blots showing the expression of acetyl-tub, HIF1α, detyr-tub, β tub and NDRG4. All proteins from Figure 7c have been analysed using the same extract (biological replicate).

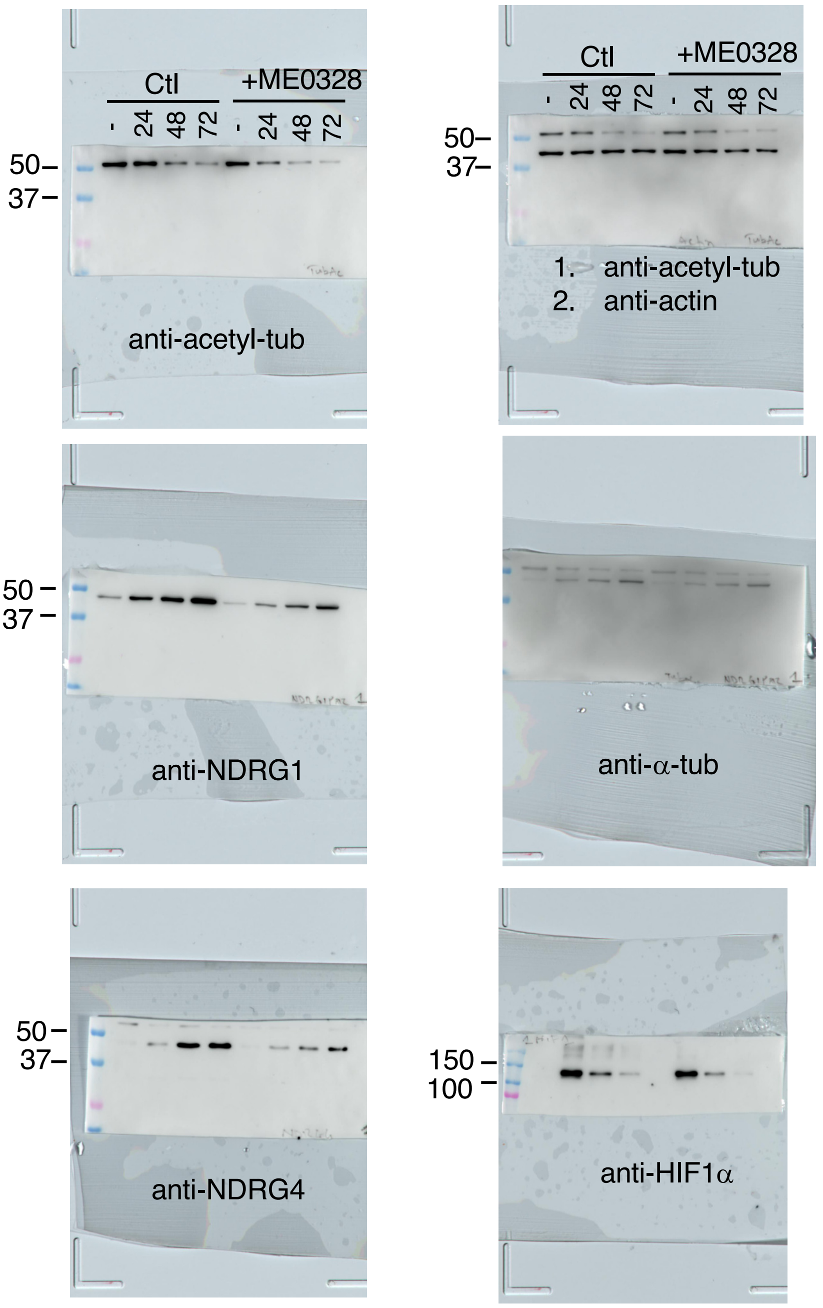

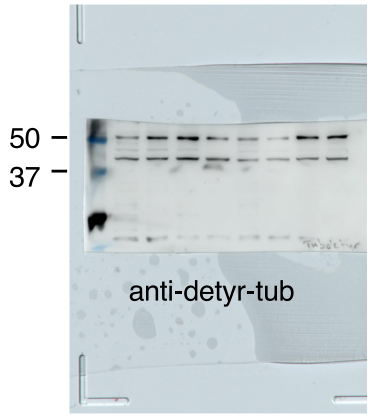

Figure 7e: Uncropped blots showing the expression of acetyl-tub, actin, NDRG1, a-tub, NDRG4, HIF1α, and detyr-tub. All proteins from Figure 7e have been analysed using the same extract (biological replicate).

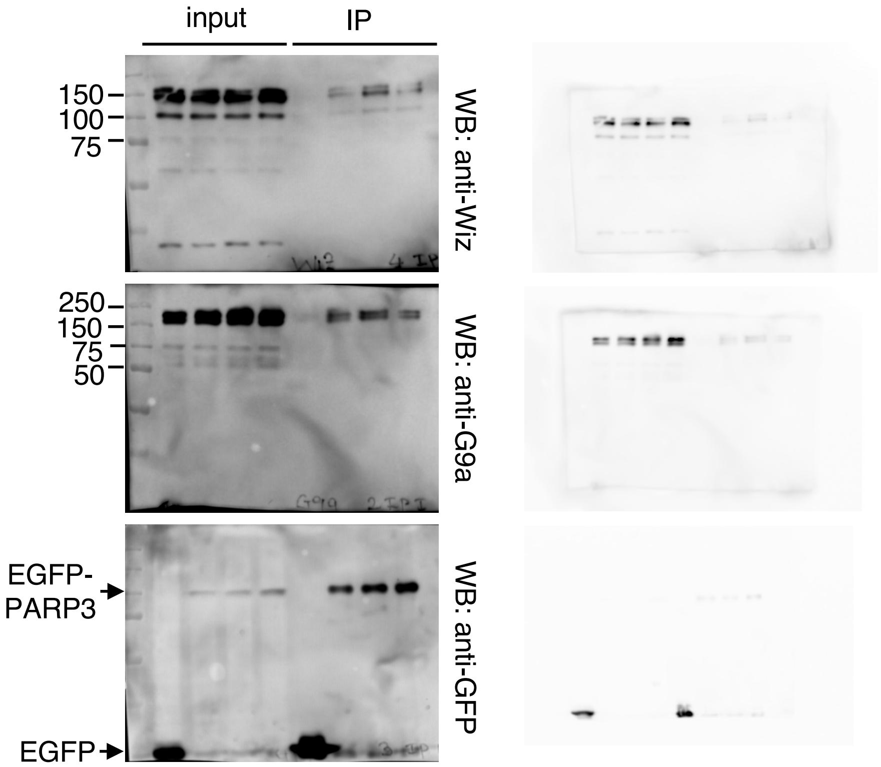

Sup. Figure 2a: Uncropped blots showing the expression of Wiz, G9a EGFP and EGFP-PARP3 in inputs and after EGFP trapping. Higher exposure times on the left side.

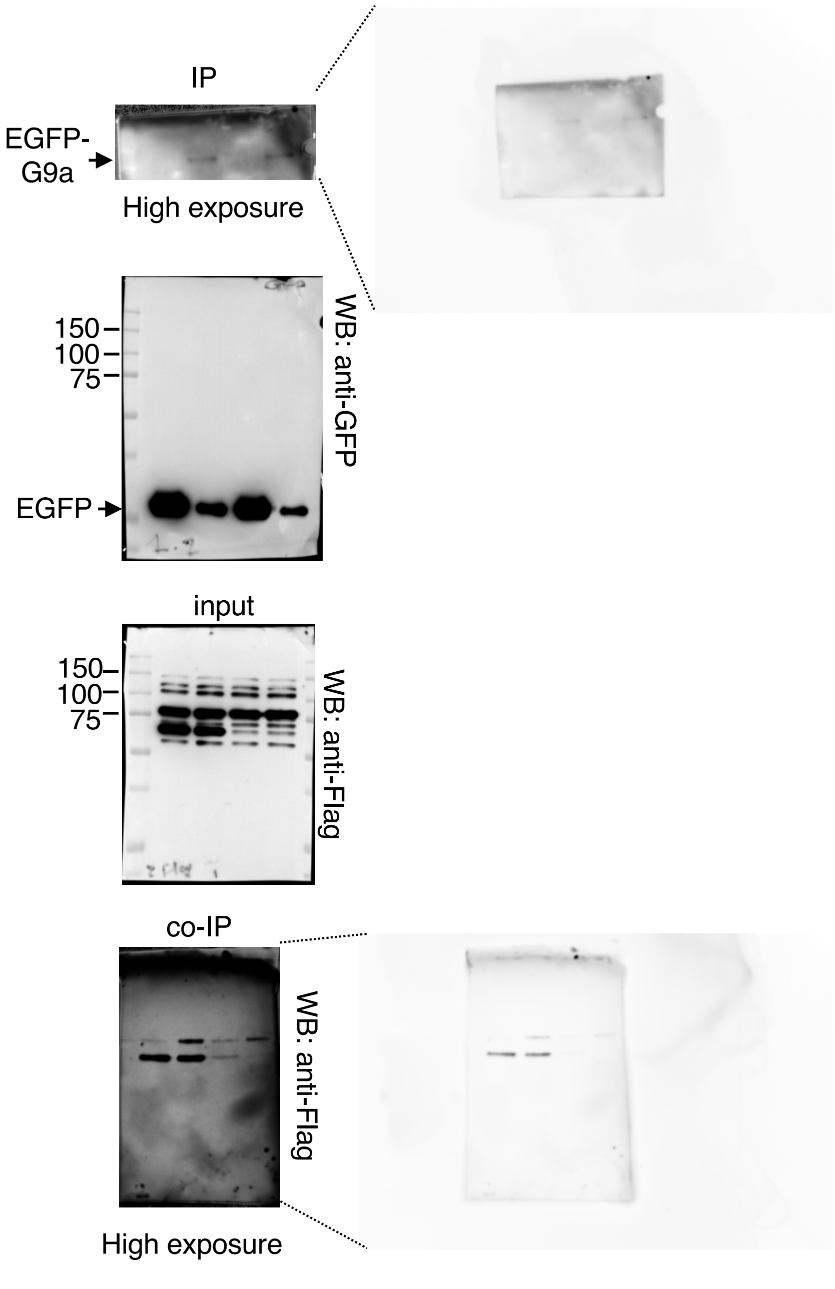

Sup. Figure 2b: Uncropped blots showing the expression of EGFP, EGFP-G9a, and Flag-PARP3 in inputs and after EGFP trapping. To reveal EGFP-G9a and Flag-PARP3, the exposure times were increased.

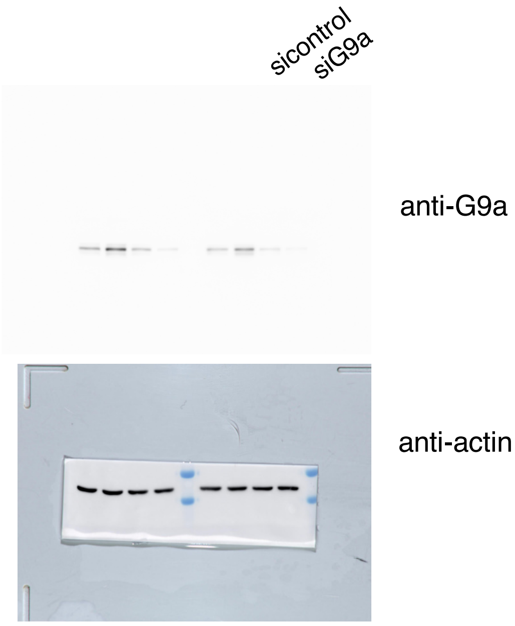

Sup. Figure 3b: Uncropped blots showing the expression of G9a and actin in T98G PARP3$^{-/-2}$ cells. Proteins have been analysed using the same extract (biological replicate). For clearer results, the membranes were cut before hybridization with antibodies.

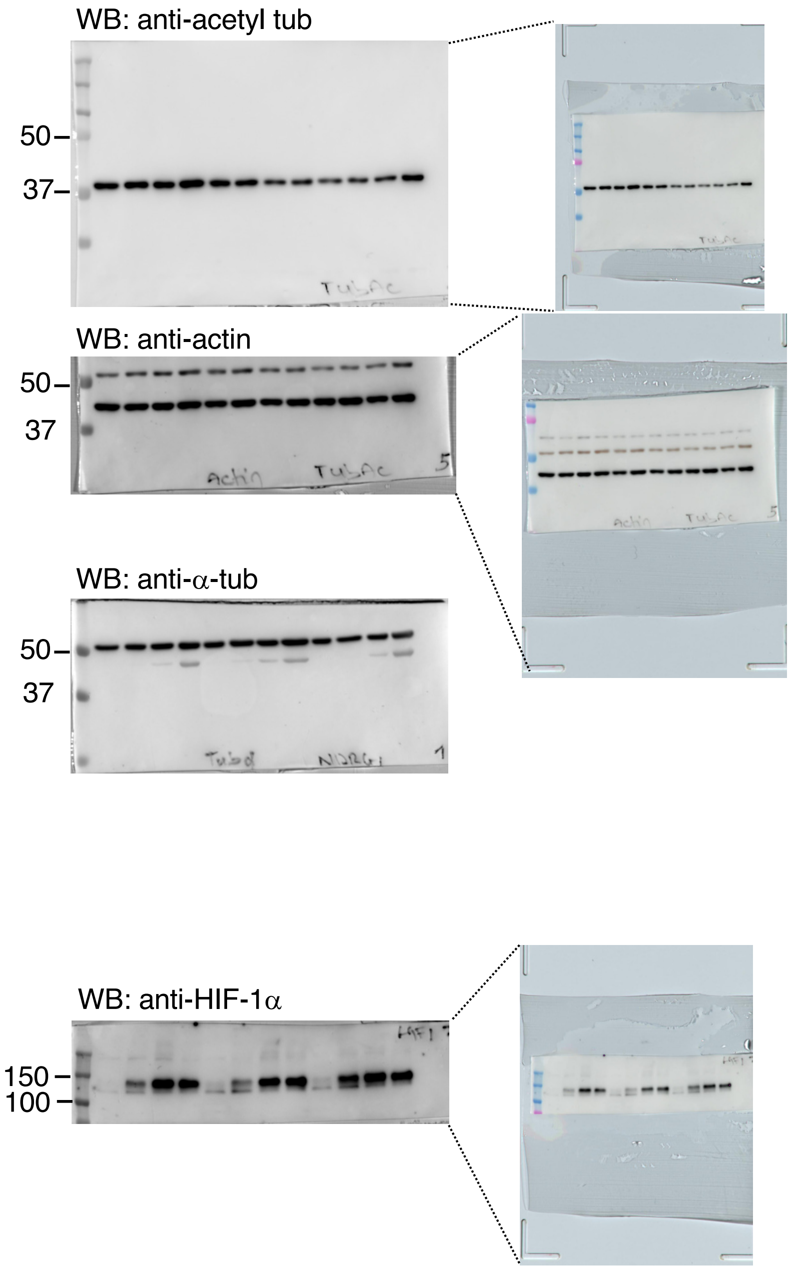

Sup. Figure 9a: Uncropped blots showing the expression of acetyl tub, actin, α-tub and HIF-1α in T98G cells. All proteins have been analysed using the same extract (biological replicate). For clearer results, some membranes were cut before hybridization with antibodies.

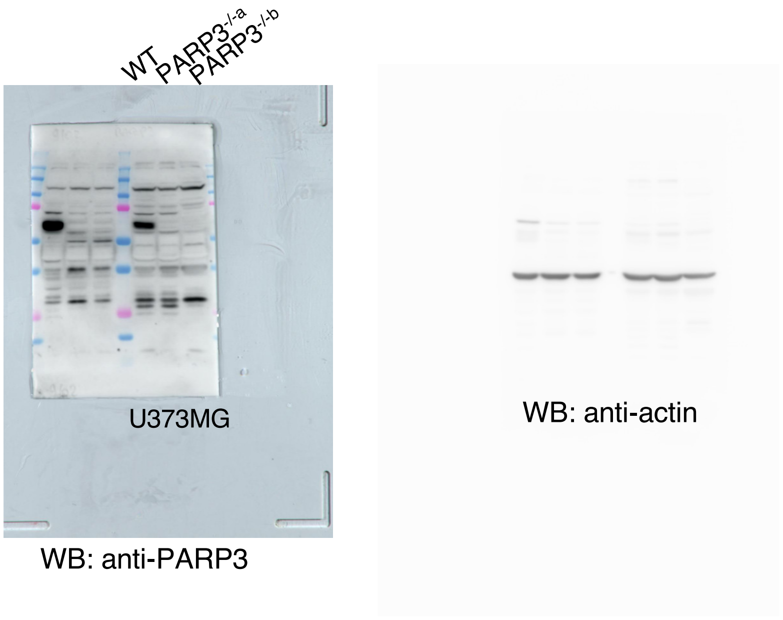

Sup. Figure 9b: Uncropped blots showing the expression of PARP3 and actin in WT and PARP3-deficient U373MG cells. All proteins have been analysed using the same extract (biological replicate).

WB: anti-NDRG4

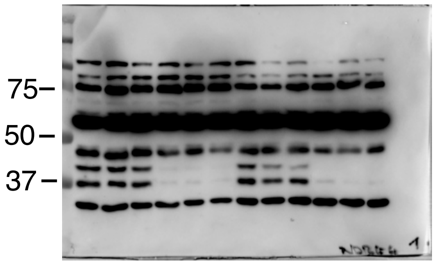

WB: anti-actin

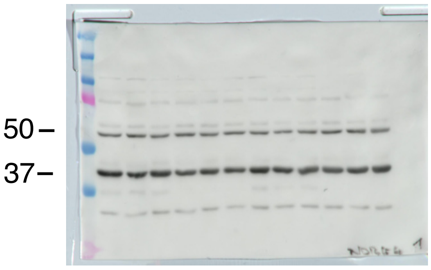

Sup. Figure 10a: Uncropped blots showing the expression of NDRG4 and actin. All proteins have been analysed using the same extract (biological replicate).
